# Lipid Scrambling Pathways in the Sec61 Translocon Complex

**DOI:** 10.1021/jacs.4c11142

**Published:** 2025-05-06

**Authors:** Matti Javanainen, Jan Šimek, Dale Tranter, Sarah O’Keefe, Sudeep Karki, Denys Biriukov, Radek Šachl, Ville O. Paavilainen

**Affiliations:** † Unit of Physics, University of Tampere, FI-33720 Tampere, Finland; ‡ Institute of Biotechnology, HiLIFE, University of Helsinki, FI-00790 Helsinki, Finland; ¶ J. Heyrovský Institute of Physical Chemistry, CZ-18223 Prague 8, Czech Republic; § Department of Physical and Macromolecular Chemistry, Charles University, Hlavova 8, CZ-12800 Prague 2, Czech Republic; ∥ Onego Bio, Hämeentie 157, FI-00560 Helsinki, Finland; ⊥ Central European Institute of Technology, Masaryk University, Kamenice 5, CZ-62500 Brno, Czech Republic; # National Centre for Biomolecular Research, Faculty of Science, Masaryk University, Kamenice 5, CZ-62500 Brno, Czech Republic

## Abstract

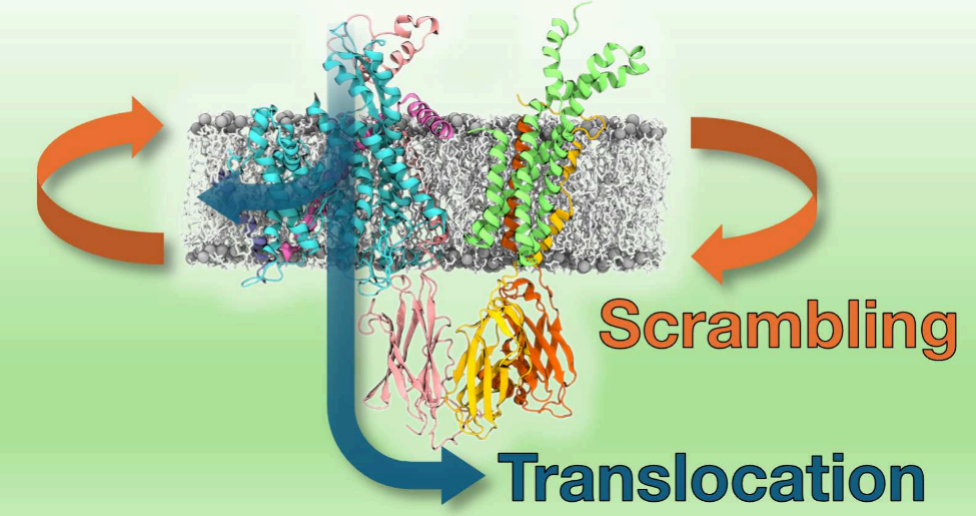

Cellular homeostasis depends on the rapid, ATP-independent translocation of newly synthesized lipids across the endoplasmic reticulum (ER) membrane. Lipid translocation is facilitated by membrane proteins known as scramblases, a few of which have recently been identified in the ER. Our previous structure of the translocon-associated protein (TRAP) bound to the Sec61 translocation channel revealed local membrane thinning, suggesting that the Sec61/TRAP complex might be involved in lipid scrambling. Using complementary fluorescence spectroscopy assays, we detected nonselective scrambling by reconstituted translocon complexes. This activity was unaffected by Sec61 inhibitors that block its lateral gate, suggesting a second lipid scrambling pathway within the complex. Molecular dynamics simulations indicate that the trimeric TRAP subunit forms this alternative route, facilitating lipid translocation via a “credit card” mechanism, using a crevice lined with polar residues to shield lipid head groups from the hydrophobic membrane interior. Kinetic and thermodynamic analyses confirmed that local membrane thinning enhances scrambling efficiency and that both Sec61 and TRAP scramble phosphatidylcholine faster than phosphatidylethanolamine and phosphatidylserine, reflecting the intrinsic lipid flip–flop tendencies of these lipid species. As the Sec61 scrambling site lies in the lateral gate region, it is likely inaccessible during protein translocation, in line with our experiments on Sec61-inhibited samples. Hence, our findings suggest that the metazoan-specific trimeric TRAP bundle is a viable candidate for lipid scrambling activity that is insensitive to the functional state of the translocon.

## Introduction

Phospholipids are the key building blocks of all cellular membranes.^1^ Cells thus need to synthesize membrane lipids to support their growth, proliferation, and homeostasis.^2^ These lipids are involved in signaling,^3^ energy storage,^4^ and membrane compartmentalization.^5^ While the majority of lipids are synthesized on the cytosolic leaflet of the endoplasmic reticulum (ER) membrane, half of newly synthesized lipids need to flip to the lumenal leaflet to eliminate differential stress.^6,7^ In general, lipids show a symmetric distribution across the leaflets of the ER membrane^8^ and are transported to the plasma membrane either along the secretory pathway, through ER–plasma membrane contact sites, or by means of nonvesicular transport.^7^ Phosphatidylserine (PS) is special, as it is exclusively located in the lumenal leaflet of the ER membrane,^8^ and its transport to the plasma membrane relies on dedicated shuttle proteins.^8^ These proteins can only pick up lipids from the cytosolic leaflet, indicating that even PS needs to occasionally cross the ER membrane to be transported.^8^

However, phospholipids contain either zwitterionic or anionic head groups, whose spontaneous permeation across the hydrophobic membrane core is energetically unfavorable. Indeed, the measured spontaneous flip–flop half-lifes for zwitterionic phosphatidylcholine (PC) lipids in fluid-phase vesicles is days to weeks.^9^ Unassisted flip–flops are therefore not likely to form the basis for maintaining lipid symmetry across the leaflets of the ER membrane. This challenge also considers other cellular membranes.^5,10^

Due to the essential biological roles of transbilayer lipid transfer and the associated substantial energetic cost, different membranes contain a suite of dedicated proteins, which significantly enhance the flip–flop rates of phospholipids. Flippases and floppases transfer lipids in specific directions against a concentration gradient by consuming ATP in the process and thus help maintain membrane asymmetry.^11−13^ Scramblases, on the other hand, increase the flip–flop rates bidirectionally without the need for energy input and thus promote leaflet symmetry.^11,12^

The role of proteins in ER membrane scrambling was already acknowledged by the 1980s,^14,15^ yet the identification of these proteins took a further three decades. During that time, it was estimated that lipid transporters constitute 0.2–1.0% of total ER membrane proteins; at least two different transporters are present; and that PC, phosphatidylethanolamine (PE), and PS are all exchanged between leaflets at the same rate, suggestive of using the same transport machineries.^16,17^

The identification of proteins capable of scrambling lipids without the help of ATP began a decade ago with opsin and TMEM16F—two proteins residing in the plasma membrane.^18,19^ Since then, the structure of a member of the TMEM16 family has also been resolved,^20,21^ allowing a description of the scrambling domain formed by hydrophilic residues in their transmembrane domain,^22^ as well as characterization of its local membrane-thinning ability.^23^ Although the exact mechanism still remains debated,^24^ these two features likely contribute to TMEM16 scramblase activity, and form the basis for our understanding of the structure–function relationship in scrambling. Very recently, the first ER-resident scramblases—TMEM41B, TMEM16K, and VMP1—were also identified.^25−30^

Lipid scrambling relies on the ability to transfer polar lipid head groups across the hydrophobic membrane core. From a physical perspective, this challenge resembles that of the integration of membrane proteins containing charged residues into the membrane or membrane translocation of charged proteins. Indeed, lipid scrambling has been very recently suggested to be a general feature of protein insertases and translocases in the ER membrane as well as in mitochondria.^31,32^

Recently, we^33^ and other teams^34−36^ resolved the structure of the translocon-associated protein (TRAP) complex associated with the Sec61 translocon in the ER membrane. Sec61 is a gatekeeper for entry of the vast majority of proteins destined for the secretory pathway. The heterotrimeric Sec61 translocon forms a transmembrane conduit through which secretory proteins enter the ER lumen and membrane proteins get integrated into the ER membrane. Sec61 assembles into various ensembles together with different auxiliary proteins to transport and coordinate processing of different nascent client proteins,^36^ with TRAP being required for translocation of certain proteins such as insulin.^37,38^

Our structural and simulation work revealed that the Sec61/TRAP complex induced local ER membrane thinning.^33^ Moreover, we noticed that the helix bundle consisting of TRAPβ, TRAPδ, and TRAPγ subunits was also rich in conserved polar amino acids. Together, these subunits form a polar groove that could potentially facilitate lipid scrambling via a similar credit card mechanism as identified for other scramblases.^32,39^ Similarly, in its open conformation, the fairly polar central channel of the Sec61 translocon could be available for the head groups of membrane lipids, although it is presumed to remain closed in the absence of an inserting polypeptide. We thus hypothesized that Sec61 and TRAP act as ER scramblases. Similar suggestions have earlier been made for the core Sec61 complex,^31,40^ and its bacterial ortholog the SecYEG translocon.^41^

To test our hypothesis, we isolated translocon complexes from sheep pancreatic ER microsomes, reconstituted the complexes into large unilamellar vesicles (LUVs), and demonstrated lipid scrambling activity using two complementary fluorescence approaches. To provide insight into the scrambling pathways, lipid selectivity, and the contribution of membrane thinning on scrambling rates, we also performed an extensive set of coarse-grained and atomistic simulations of membrane-embedded Sec61/TRAP complexes. Our experiments with Sec61 inhibitors that block the central channel suggest an alternative scrambling pathway within the translocon, and our simulations suggest that this conduit could be formed by the trimeric transmembrane bundle of the TRAP complex.

## Results and Discussion

### Fluorescence Assays Demonstrate Scrambling by the Translocon Machinery

**Figure 1:**
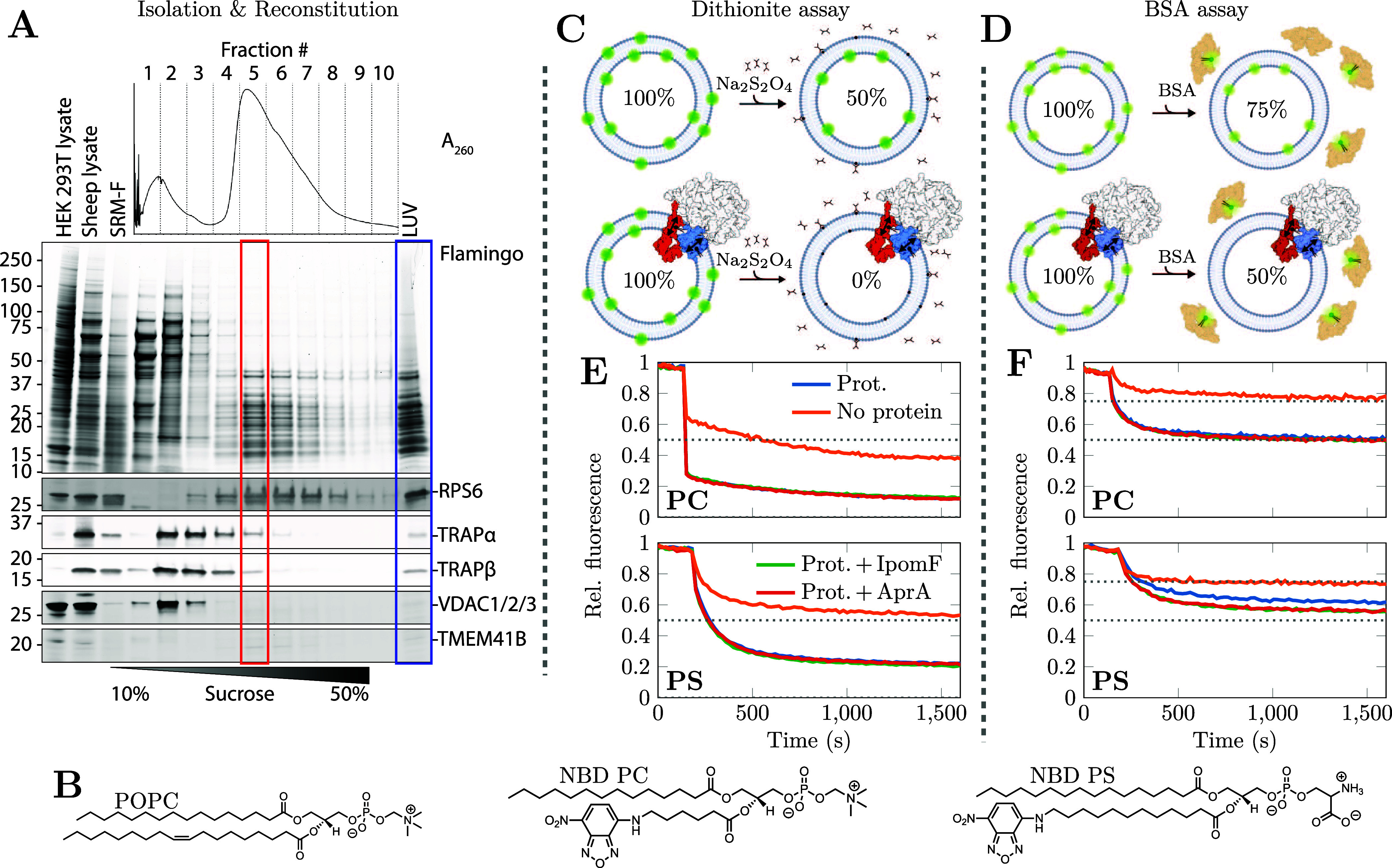
Translocon reconstitution and fluorescence assays. A) Sucrose gradient purification of detergent-solubilized ribosome/Sec61 translocon complexes. A_260_ UV trace (top), total protein staining (middle), and Western blot analysis (bottom) of the sucrose gradient fractions. HEK293T and sheep whole pancreatic lysates are included as antibody controls. The fraction used in the reconstitution (red) is highlighted together with the composition of the LUVs (blue). The uncropped gels are available in Figure S1 in the SI. B) Chemical structures of the lipids used in experiments. The LUVs are made of POPC with 0.4% (mol) of *sn*-2 acyl chain-NBD-labeled PC (NBD PC) or PS (NBD PS). C) The sodium dithionite, added to the supernatant, chemically reduces the fluorescent NBD PC or NBD PS to nonfluorescent ABD PC or ABD PS in the outer leaflet. This leads to a decrease of fluorescence intensity by ≈50% of its initial value.^44^ In the presence of a scramblase, all NBD-labeled lipids eventually reach the outer leaflet resulting in an almost complete loss of NBD fluorescence. D) The addition of bovine serum albumin (BSA) to the sample leads to partial quenching (≈50%) of NBD fluorescence. Hence, in the absence (presence) of a scramblase, a decrease of ≈25% (≈50%) is expected. E) Results from the dithionite assay. Fluorescence before dithionite addition is normalized to 1, and the curves show a mean of *N* = 2 repeats. Dashed line shows the expected result in the case of no scrambling (0.50), and further decrease is likely due to the dimming of NBD over time. In the absence of proteins, the NBD PC and NBD PS in the outer leaflet is chemically reduced, resulting in a fluorescence intensity decrease to ≈50% of the original value. In the presence of proteins, the value further decreases to ≈15%, i.e. the majority of the NBD-labeled lipids originally in the inner leaflet are scrambled to the outer leaflet and reduced therein. The presence of Sec61 inhibitors (here either Ipomoeassin F, “IpomF” or Apratoxin A, “AprA”) do not affect scrambling. Overall, the interaction between NBD PS and dithionite seems slower, likely due to the electrostatic repulsion between anionic PS head groups and dithionite. F) Results from BSA assay, confirming the findings of panel E). The dashed lines shows the expected results in the case there is (0.50) or is not (0.75) scrambling. The curves show mean of *N* = 2 repeats. The presence of Sec61 inhibitors (IpomF or AprA) had no effect on scrambling. Some graphical elements in panels C and D were created with BioRender.com, and they are available online: Šimek, J. (2025)https://BioRender.com/sx9c95m.

The Sec61/TRAP complex induces local membrane thinning,^33^ and contains potential scrambling pathways lined with polar residues in each of its constituents, therefore rendering it a potential ER lipid scramblase. To test this hypothesis, we isolated the ribosome–translocon machinery from purified rough ER microsomes, from which we have previously purified ribosome/Sec61/TRAP complexes for cryo-EM studies.^33,42^ Sucrose gradient centrifugation enabled efficient separation of solubilized ribosome/Sec61 complexes from contaminating nonribosome associated proteins at a scale required for LUV preparation. A single fraction was used for reconstituting the ribosome/Sec61 translocon complexes into large unilamellar vesicles (LUVs) made of 1-palmitoyl-2-oleoyl-*sn*-glycero-3-phosphocholine (POPC) lipids which also contained NBD-labeled phosphatidylcholine (NBD PC) or phosphatidylserine (NBD PS) (Figure 1B). The distribution of ribosome/Sec61 translocon complexes in sucrose gradient and the presence of ribosome an Sec61 subunits in the resulting reconstituted LUVs were confirmed by Western blotting (see Figure 1A) and mass spectrometry (see Supporting Information (SI)). Collectively, the quantitative Western blot and mass spectrometry analyses support high level of enrichment of ribosome/Sec61/TRAP in the peak fraction from the known scramblases VDAC1–3 and TMEM41B.^26−32,43^

A dithionite assay (Figure 1C) demonstrated lipid scrambling activity for both NBD PC and NBD PS in the LUVs containing reconstituted translocons. In our control system, lipid-only LUVs, dithionite chemically reduced only ≈50% of fluorophores (Figure 1E). This indicates that dithionite could only interact with the NBD groups attached to the acyl chains of the lipids in the outer leaflet of the LUVs, in line with the absence of scrambling activity in the timeframe of the experiment. However, in LUVs with reconstituted proteins, fluorescence loss was nearly complete for both NBD PC and NBD PS. This indicates that either dithionite was able to penetrate into the vesicles, or the lipids from the inner leaflet were scrambled to the outer one where their NBD labels were accessible to dithionite.

To rule out dithionite permeating into LUVs, we also performed the assay using bovine serum albumin (BSA) as the quenching agent (Figure 1D). BSA is significantly bulkier than dithionite and hence cannot enter the interior of intact LUVs passively or through membrane pores. The scrambling of both NBD PC and NBD PS in LUVs with reconstituted proteins was again observed as BSA quenched essentially all accessible NBDs, resulting in a decrease of fluorescence to ≈50% of its value before BSA addition (Figure 1F). This is in line with the fact that BSA only quenches a maximum of 50% of NBD fluorescence.^17,45^ Again, in the lipid-only control LUVs, BSA accessed only the lipids in the outer leaflet, and hence a decrease to ≈75% of the original signal was observed.

**Figure 2:**
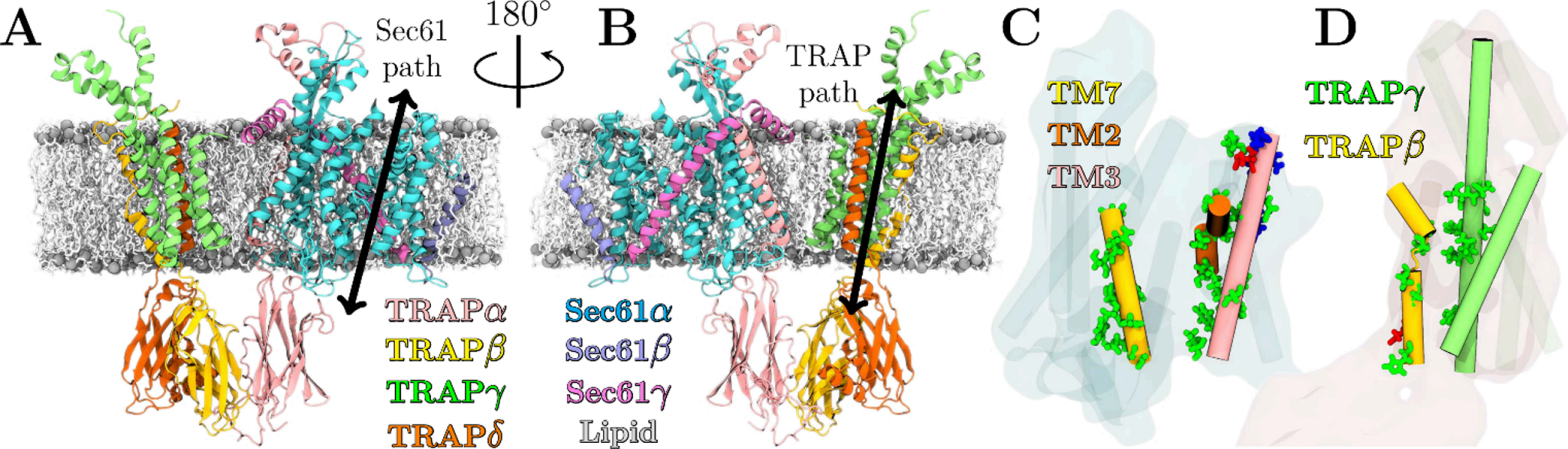
Scrambling pathways of the Sec61/TRAP complex. A and B) Snapshots of the Sec61/TRAP complex from A) the side of the lateral gate, “front” and B) from the opposite side, “back”. The prospective scrambling paths of Sec61 along the lateral gate and of TRAP along the groove between the TRAPβ and TRAPγ are highlighted by bidirectional arrows. The structure is embedded in a lipid bilayer shown in gray (phosphorus atoms) and white (rest) to highlight the transmembrane regions. C and D) The polar (green), anionic (red), and cationic (blue) residues located near the potential scrambling pathways are highlighted for C) Sec61 and D) TRAP.

To test whether blocking the Sec61 central pore would influence the observed lipid scrambling activity, we repeated both the dithionite and BSA assays in the presence of two small molecule Sec61 inhibitors that bind the lateral gate region and thereby prevent protein translocation.^46^ We speculated that an inhibitor-mediated blockade would also prevent Sec61 from scrambling lipids since the translocation and scrambling take place during the same pathway. We used Ipomoeassin F^47^ and Apratoxin A.^48^ Importantly, both inhibitors were used at a saturating concentration of 1 μM (IC_50_ for both compounds is in the 100 nM range^47,48^) and therefore we expect the aqueous channel and lateral gate of Sec61 to be fully occluded during the experiment. Apratoxins and Ipomoeassin F bind to the same region of the Sec61 lateral gate,^46^ yet the gate obtains a more closed conformation with the latter, rendering the polar residues (Figure 2C) largely inaccessible for lipid head groups. The addition of either inhibitor had no observable effect on lipid scrambling (Figures 1E and 1F), suggesting existence of a distinct scrambling pathway independent from the lateral gate of Sec61. However, due to the potential presence of other translocon components beyond Sec61 and TRAP in nonstoichiometric ratios,^36^ we cannot pinpoint this activity to a specific protein based on the experimental results alone.

### Simulations Identify TRAP as a Putative Translocon-Associated Lipid Scrambling Factor

Due to the inherent challenges in generating purified samples of Sec61/TRAP without other auxiliary translocon components, we next leveraged molecular dynamics simulations to investigate the mechanism of lipid scrambling by Sec61 and TRAP. These two proteins form the central stable core of the translocon machinery,^36^ and TRAP is a viable candidate for the scrambing activity observed in our experiments with Sec61 inhibitors: It contains the suitable polar transmembrane crevice, it stably associates with the translocon,^49^ and we detected its presence in our LUVs, Figure 1A.

We used our recently resolved model for the core Sec61/TRAP complex bound to the mammalian ribosome.^33^ Since some of the extramembrane parts and flexible loops could not be unambiguously determined based on cryo-EM in our model (PDB: 8BF9), they were built using MODELER^50^ for this work (see Figure 2A and B). Notably, our experimental sample also contained a substrate-selective Sec61 inhibitor KZR-8445 that, together with the ribosomal docking to Sec61, maintained the Sec61 lateral gate in an open conformation. Therefore, the gate is also open in our model.^42^

The prospective scrambling pathways in Sec61 and TRAP are highlighted in Figure 2A and B, respectively. We predict these pathways based on the presence of groove-like structures containing polar residues that could shield the anionic or zwitterionic lipid head groups as they traverse the hydrophobic core of the membrane. The Sec61 pathway is used for the translocation of nascent polypeptides, so the lining of the lateral gate region by polar residues is not surprising (Figure 2C). However, the bundle formed by the three TRAP subunits also contains a significant amount of polar residues in its transmembrane segments: TRAPγ has numerous serines and asparagines in its four-helix transmembrane bundle, whereas TRAPβ contains a serine and a threonine in the very core of the membrane around its helix-breaking P158 (Figure 2D).

In order to sample time scales required for spontaneous lipid scrambling, we performed a resolution transformation of our Sec61/TRAP model into the Martini 3 coarse-grained force field.^51^ We performed simulations with either the complete Sec61/TRAP complex, the isolated trimeric Sec61 complex, or the tetrameric TRAP complex embedded in a POPC bilayer (Set 1 in Table 1). The functional Sec61/TRAP complex is locked into a specific conformation by ribosomal anchoring,^33^ which we opted to model by restraining the protein backbone in the simulations. This also means that the lateral gate of Sec61—opened by the inhibitor^33,42^—remains open throughout the simulations. All simulations, listed in Table 1, were performed in five replicates for 20 μs each using the recommended simulation settings^51,52^ (see Methods for details).

**Table 1:** Simulated Systems and the Observed Flip–Flop Rates^a^

System	*k*_PC_	*k*_PE_	*k*_PS_	*k*_tot_
**1: Single-component membrane**				
Sec61/TRAP^b^	7.7 ± 0.7	–	–	7.7 ± 0.7
Sec61	6.5 ± 1.8	–	–	6.5 ± 1.8
TRAP	2.2 ± 0.3	–	–	2.2 ± 0.3
Sec61/TRAP + NaCl	1.6 ± 0.3	–	–	1.6 ± 0.3
Sec61 + mutations	14.0 ± 0.4	–	–	14.0 ± 0.4
**2: Multicomponent membrane**				
Sec61/TRAP	2.6 ± 0.3	0.6 ± 0.7	1.0 ± 0.2	4.2 ± 0.4
Sec61	2.8 ± 0.4	0.8 ± 0.2	1.2 ± 0.5	4.8 ± 0.8
TRAP	0.7 ± 0.2	0.0 ± 0.0	0.2 ± 0.1	1.0 ± 0.1
**3: TRAP subunits, multicomponent membrane**				
TRAPα	0.0 ± 0.0	0.0 ± 0.0	0.0 ± 0.0	0.0 ± 0.0
TRAPβ	0.0 ± 0.0	0.0 ± 0.0	0.0 ± 0.0	0.0 ± 0.0
TRAPγ	0.1 ± 0.1	0.0 ± 0.0	0.0 ± 0.0	0.1 ± 0.1
TRAPδ	0.0 ± 0.0	0.0 ± 0.0	0.0 ± 0.0	0.0 ± 0.0
**4: Sec61/TRAP complex, varying temperature**				
290 K	1.7 ± 0.4	–	–	1.7 ± 0.4
300 K	3.3 ± 0.6	–	–	3.3 ± 0.6
310 K^b^	7.7 ± 0.7	–	–	7.7 ± 0.7
320 K	14.3 ± 1.1	–	–	14.3 ± 1.1
330 K	26.0 ± 1.0	–	–	26.0 ± 1.0

a The rates are reported for different lipid types (*k*_PC_, *k*_PE_, and *k*_PS_), and the overall rate (*k*_tot_) is also provided, all in 1/μs. The single-component membrane was made up of POPC, whereas the multi-component one consisted of POPC, 1-palmitoyl-2-oleoyl-*sn*-glycero-3-phosphoethanolamine (POPE), and 1-palmitoyl-2-oleoyl-*sn*-glycero-3-phosphoserine (POPS) (see Methods for details). The mean values are calculated from five (four for the Sec61 system with mutations) 20 μs-long replica simulations and shown together with the standard error. The total simulation time is ≈1.5 ms.

b Same simulation.

Visual observation of the simulations immediately confirmed our experiment-backed hypothesis that lipid scrambling takes place in the immediate vicinity of the Sec61/TRAP complex, where significant membrane remodeling was observed by cryo-EM.^33^ To quantify the scrambling events between the leaflets, we traced the lipid orientations (see Methods). The rates (in lipids/μs) of PC lipids scrambled by the different complexes are listed in the first section of Table 1. From this analysis, it is evident that both Sec61 and TRAP scramble PC lipids at a rate exceeding one lipid per microsecond. This is in the same order of magnitude as the >100,000 lipids scrambled per second *in vitro* by opsin, a well-studied plasma membrane scramblase.^53^ In our simulations, the scrambling by Sec61 is some ≈3-fold faster than that of TRAP (*p* = 0.0009), yet both contribute at physiologically relevant rates. A similar ratio is also found in the Sec61/TRAP system if the flip–flops are assigned to the two scrambling sites based on distance. The Sec61/TRAP complex scrambles lipids at a rate similar to the sum of the two complexes placed in the membrane alone (*p* = 0.31), indicating that there are no synergistic or antagonistic effects for Sec61 and TRAP. In fact, the scrambling rate of the Sec61/TRAP complex is not significantly larger than that of Sec61 alone in our simulations (*p* = 0.19).

### Lipids are Scrambled via the Credit Card Mechanism

**Figure 3:**
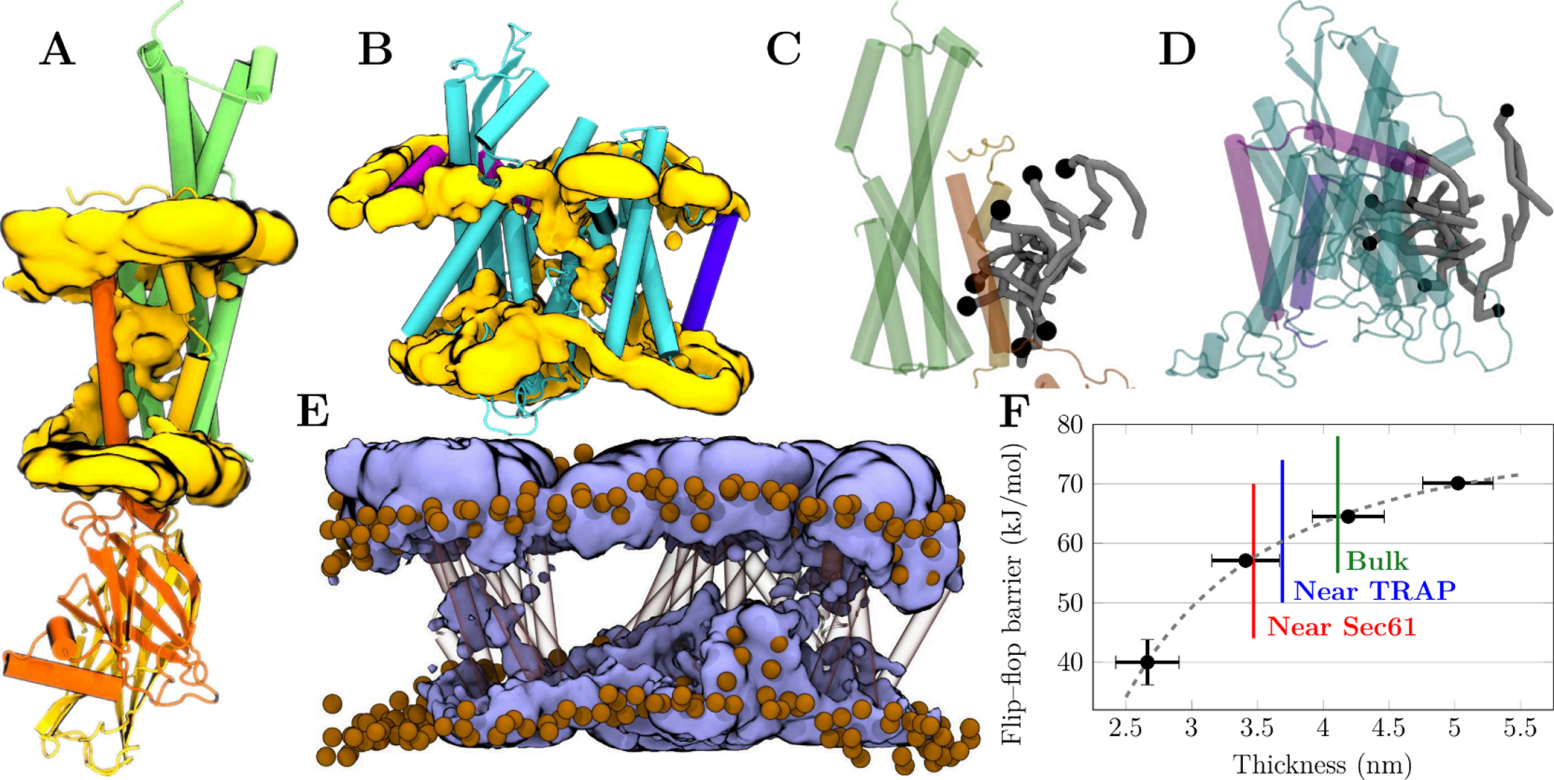
Mechanism of lipid scrambling by Sec61/TRAP. A and B) Volumetric density maps of the lipid headgroup phosphate beads (“PO4”) within A) the bundle of transmembrane helices of the TRAPβ, TRAPγ, and TRAPδ subunits or B) Sec61. Averaged from five 20 μs-long simulations of the Sec61/TRAP complex in the multicomponent membrane. TRAP and Sec61 subunits are colored as in Figure 2. The densities of the different lipid types are provided in Figures S2 (Sec61) and S3 (TRAP) in the SI. For visualization, atomistic protein structures are employed in the rendering. C and D) Snapshots of scrambling mechanism by C) the bundle of transmembrane helices of the TRAPβ, TRAPγ, and TRAPδ subunits or D) Sec61. The lipid headgroup (black bead) partitions to the polar crevices highlighted in Figure 2C and 2D, whereas the acyl chains (gray) remain in the membrane environment. Coloring of subunits as in Figure 2 and panels A and B. For visualization, atomistic protein structures are employed in the rendering. E) Volumetric density maps of water from atomistic simulations of the Sec61/TRAP complex. Water density is shown in blue, protein in transparent surface with TRAPβ, TRAPγ, and TRAPδ on the left and Sec61 with TRAPα on the right. Lipid headgroup phosphorus atoms are shown as brown spheres to highlight membrane thickness and curvature perturbations. F) Effect of membrane thickness on the free energy barrier for lipid flip–flop. Black markers show thickness/barrier pairs calculated from a set of bilayers comprised of lipids with saturated chains of varying length, whereas the gray dashed line is provided as a guide to the eye. The colored lines show the thickness values observed in the vicinity of Sec61, in the vicinity of the bundle of TRAPβ, TRAPγ, and TRAPδ subunits, and far away from the proteins in the “bulk” membrane. The thinning induced by the proteins lowers the free energy barrier by an estimated ≈4.1 kJ/mol (TRAP) or ≈6.8 kJ/mol (Sec61). These values are extracted as the differences of the intersections of the colored lines and the gray dashed fit along the *y* axis.

Visual inspection of the simulation trajectories revealed that the lipid flip–flops take place through a crevice formed by either the helices of the trimeric TRAP complex or the lateral gate of Sec61 (Figures 2C and 2D). These pathways are also quantified by volumetric densities of the lipid headgroup beads, extracted from simulations of the Sec61/TRAP complex in a multicomponent membrane containing equimolar amounts of POPC, POPE, and POPS (Set 2 in Table 1). These densities are visualized in Figures 3A and 3B for TRAP and Sec61, respectively. They reveal significant and continuous populations of the lipid head groups in the membrane and between the prospective scrambling pathways shown in Figure 2C and 2D. The selected events visualized in Figure 3C and 3D for Sec61 and TRAP demonstrate that lipids are scrambled via a credit card mechanism,^32,39,54^ in which the polar headgroup of the lipid traverses the membrane interior within the polar crevice, whereas its acyl chains remain in the hydrophobic membrane environment. This way, interactions between polar and nonpolar environments are avoided without energy input.

Lipid scrambling by Sec61 is not entirely surprising, considering that the simulations are performed using the open conformation of the Sec61 channel.^33,42^ The open lateral gate provides an extensive trans-bilayer pathway that simultaneously fits multiple lipid head groups and can thus scramble multiple lipids simultaneously, even in opposite directions. This scrambling ability of Sec61 complexed with oligosaccharyl transferase and TRAP was also recently observed in another study using coarse-grained simulations,^31^ and the role of the lateral gate conformation was found to be crucial; with a closed gate, the scrambling ability was largely suppressed. Our analysis indeed found no alternative pathways for scrambling on the Sec61 surface, as evidenced by the lack of continuous interleaflet headgroup densities in Figure S2. Still, in our structure, the plug helix and its charged loops somewhat occlude the lateral gate region, and perhaps in another conformation of this region, scrambling could be even faster than observed here.

### TRAP Scrambles More Lipids Under Physiological Conditions

Under physiological conditions, Sec61 translocon pore remains closed in order to block undesired calcium leakage from the ER lumen, and therefore it seems unlikely that Sec61 would play a major role in bulk lipid scrambling in a physiological setting. In line with this, Vehring et al.^40^ observed an insignificant effect on lipid scrambling in yeast ER membranes upon the depletion of Sec61p.^40^

In its closed conformation, the plug helix and the closed lateral gate seal the lumenal end of the Sec61 channel.^55^ It is only upon docking of the ribosome onto Sec61 that the gate partially opens (“priming”),^56^ whereas the association of the signal peptide renders the gate fully open.^57^ Thus, Sec61 may never reside in a state with its lateral gate open and not occupied by a nascent polypeptide being translocated by Sec61. Alternatively, the gate can also be opened by Sec61 inhibitors,^42,46,58^ yet they also associate with the polar residues of the lateral gate along the scrambling pathway. One possibility is that the presence of TRAP and the ribosome promote a more open conformation of the lateral gate and thus assists in initiating the translocation of certain proteins.^33,37^ This could provide a time window for Sec61 to scramble lipids.

Identification of the Sec61 lateral gate as a lipid scrambling site, and the more general view of protein insertases acting as scramblases,^31^ prompts the question whether the lipid scrambling and polypeptide insertion/translocation activities could be functionally linked. Both processes involve the passage of charged groups—be it lipid head groups or charged protein residues—through the hydrophobic core of a membrane. As a first attempt to uncouple each function, we considered a set of residues; I41, D60, M65, R66, S71, G80, S82, T86, Y131, and M136, whose mutations are known to provide resistance for Sec61 inhibitors yet not affect nascent polypeptide translocation in the absence of an inhibitor.^59^ Hence, if their mutation into alanines inhibited scrambling, it would indicate that scrambling and translocation could indeed be uncoupled. However, we observe that these mutations actually *increased* scrambling rates for Sec61 in the POPC membrane (*p* = 0.0001, see “Sec61 + mutations” in Table 1). Hence, more experimental data on translocation- or scrambling-inhibiting mutations are required for the conclusive evaluation of the interconnection of these two processes.

Curiously, the salt present in the simulations also plays a role in determining scrambling rate. The majority of our simulations were performed only with counterions necessary to neutralize excessive protein charges, but we also repeated the simulation of the Sec61/TRAP complex in the presence of 150 mM NaCl in the aqueous phase (last entry of Set 1 in Table 1). In five replica simulations, each 20 μs long, the ions crowd the cytosolic vestibule of the Sec61 channel and thereby inhibit lipid scrambling. Indeed, when we assign flip–flops in this system to either the Sec61 or TRAP complex, we find that TRAP scrambles more lipids in the presence of salt (*p* = 0.0005), indicating a drastic change from the ≈3-fold faster rate observed for Sec61 compared to TRAP observed in the absence of ions. This effect of salt is also corroborated by the volumetric densities of lipid headgroup beads in Figure S5, which demonstrate that in the presence of salt, the continuous density bridging the membrane leaflets is lost at the site of Sec61, whereas it is present in the absence of salt. The observed strong tendency for the monovalent ions to crowd the entry point into the Sec61 channel highlights how scrambling can be sensitive to ambient conditions and that Sec61 activity might be especially affected.

These observations question the role of Sec61 as a physiologically relevant scramblase. Still, TRAP is present in a substantial majority of all ribosome-associated Sec61 complexes,^36^ and it could thus provide a means for lipid scrambling regardless of the functional state of the translocon. Hence, TRAP present in our LUV reconstitutions (Figure 1A) could be behind the experimentally observed scrambling of the reconstituted translocon machinery, and exclusively so in the presence of a Sec61 inhibitor (Figures 1E and 1F). Moreover, TRAP scrambling activity is unaffected by the presence of salt in simulations. This is supported by the volumetric density maps in Figure S5, which demonstrate a continuous density of lipid head groups along the TRAP scrambling pathway regardless of the presence of ions. A plausible explanation for this phenomenon is that, in contrast to Sec61, the transmembrane region of TRAP lacks charged residues, which may attract ions that block the scrambling pathway.

To further look into the structural features behind the scrambling ability of TRAP observed in simulations, we also simulated each of the four individual TRAP subunits alone (Set 3 in Table 1). In these simulations (5 replicas for each subunit, 20 μs per replica), we only observed a few scrambling events for TRAPγ. Moreover, these simulations were performed on a multicomponent membrane containing POPC, POPE, and POPS, indicating that none of these lipid moieties is scrambled efficiently by the individual TRAP subunits. These data indicate that TRAP complex-mediated lipid scrambling in our simulations arises from cooperation between the subunits. Since the TM domain of TRAPα is located far away from the TM domains of other TRAP subunits, it does not contribute to the scrambling ability, and therefore, all of the potential activity must arise from the six transmembrane domains in the bundle of the TRAPβ, TRAPγ, and TRAPδ subunits.

### Polar Intra-Membrane Residues and Membrane Thinning Facilitate Scrambling

Both a membrane-spanning pathway of polar residues as well as local membrane thinning have been suggested to contribute to scrambling activity.^22,23^ In line with this, simulations have demonstrated how lipid-lined pores also form easier in thinner model membranes.^60^ While we observed lipid scrambling consistently in our simulations, it remains possible that our coarse-grained description omits some key structural or chemical details. Thus, we turned to all-atom simulations of the Sec61/TRAP complex in a POPC membrane to look into these features. While the structure is independent of the resolution used in the simulations, it is unclear whether the identified scrambling sites (Figures 2C and 2D) are polar enough to host the lipid head groups. To this end, we calculated the volumetric density map of water molecules from the all-atom simulations. The map in Figure 3E demonstrates that a significant amount of water penetrates into the membrane interior at both scrambling sites, supporting partitioning of the polar head groups therein. The limited time scale and a more rugged free energy landscape unfortunately preclude the observation of spontaneous lipid flip–flops in atomistic simulations.

Regarding local membrane thinning, our earlier all-atom simulations and analysis of Sec61/TRAP in native membranes^33^ already suggested that the membrane is thinner in the vicinity of Sec61 and TRAP. However, it is unclear how much this thinning contributes to the scrambling activity. To tackle this question, we performed biased accelerated weight histogram (AWH) simulations on a set of PC lipids with saturated acyl chains of increasing lengths (see Methods). We then extracted the free energy barriers and membrane thicknesses from these simulations to estimate the trend between these two quantities. Next, we extracted membrane thickness values from the Sec61/TRAP simulations in the multicomponent membrane (Set 2 in Table 1). We classified the lipids in proximity to Sec61 (a headgroup within 2 nm of any Sec61 subunit) or near TRAP (a headgroup within 2 nm of the TRAPβ, TRAPγ, or TRAPδ subunit), or far from the translocon complex (headgroup not within 3 nm of any protein subunits). The thicknesses calculated for these lipid populations are shown in Figure 3F as colored lines. The local thinning due to the presence of the Sec61/TRAP complex seems to contribute to the lowering of the free energy barrier by ≈7 kJ/mol at most, in case the trend observed for the set of lipids with saturated acyl chains holds. Although it might seem minor in the protein-free case, such a decrease can easily render lipid scrambling thermally accessible if the other mechanism—polar intramembrane residues—has brought it down to a suitably low level.

### Enthalpic Gain Dominates Over the Loss in Entropy in Scrambling

**Figure 4:**
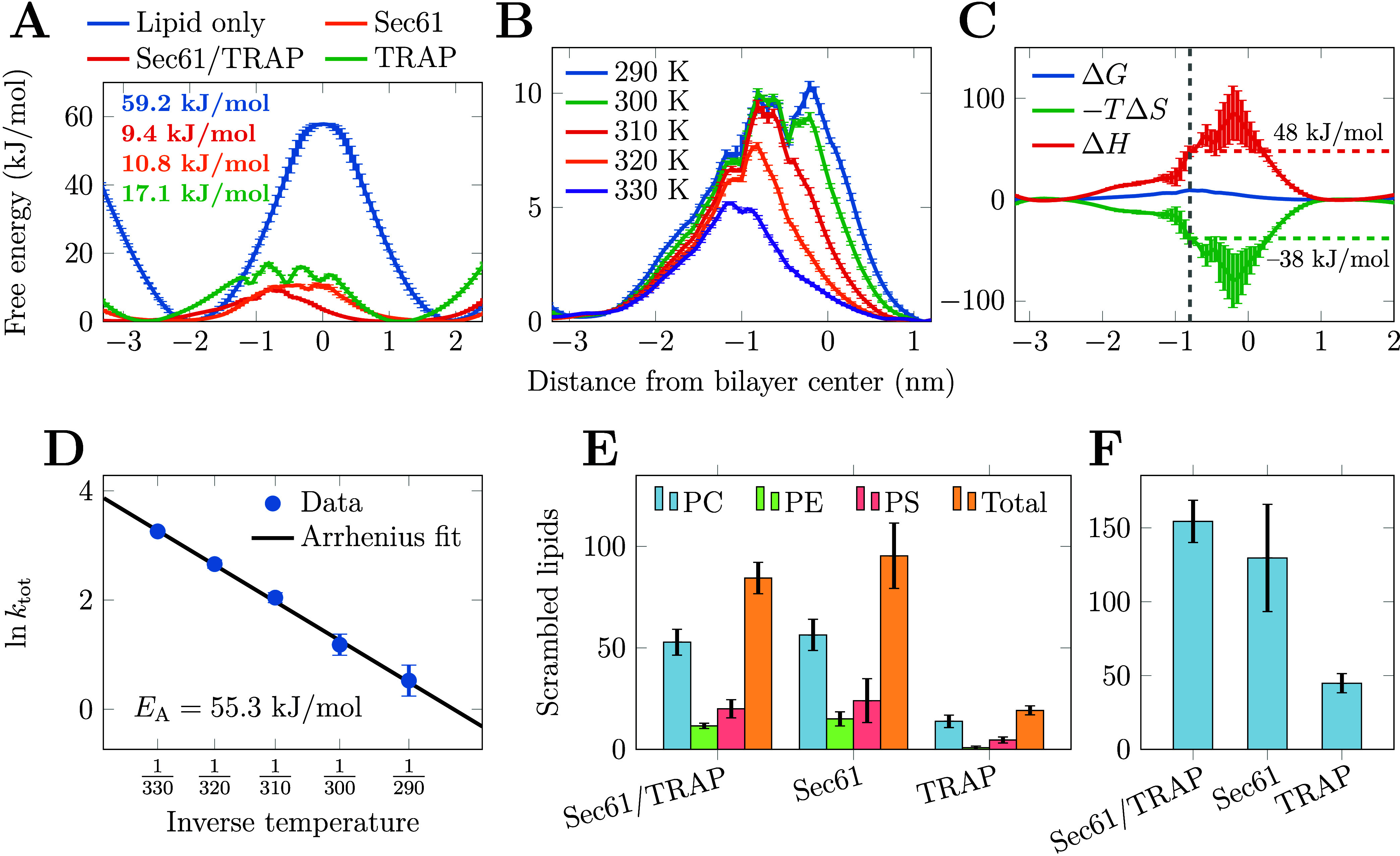
Energetics of lipid scrambling by Sec61/TRAP. A) The free energy profiles for a POPC flip–flop in the single-component membrane in the presence of Sec61, TRAP, or the Sec61/TRAP complex as well as in the protein-free membrane at 310 K. The profiles are calculated from density profiles and using biased AWH simulations, respectively (see Methods). Error bars are calculated from the difference of the profiles in the two membrane leaflets (protein-free system) or as the standard deviation of the five replica simulations (protein-containing systems). B) Temperature dependence of the free energy profile for lipid scrambling by Sec61/TRAP in the single-component membrane (Set 4 in Table 1). The profiles are calculated from density profiles (see Methods). The error bars show the standard deviation of the five replica simulations. C) Decomposition of the free energy profile into entropic and enthalpic components performed by assuming a constant enthalpy in the simulated temperature range (see Methods). The error bars show the difference between two estimates calculated from two pairs of temperatures (300 K and 320 K as well as 290 K and 330 K, Set 4 in Table 1). The location of the barrier at 310 K is marked with a dashed gray line, and the corresponding entropic and enthalpic values are also indicated. D) Arrhenius analysis of lipid scrambling rate by Sec61/TRAP in the POPC membranes (Set 4 in Table 1). The scrambling rates observed at different temperatures are fitted with , from which the activation energy *E*_A_ is obtained. The error bars show relative error, yet they are not visible for most of the data points. E) Lipid headgroup selectivity of the scrambling activity of Sec61, TRAP, and Sec61/TRAP complexes. Bars show the mean and standard error for the number of total scrambled lipids extracted from the five 20 μs-long replica simulations of the multicomponent membrane (Set 2 in Table 1). The membranes contain equal amounts of PC, PE, and PS. F) Scrambling of POPC by Sec61, TRAP, and Sec61/TRAP complexes in the single-component membrane (Set 1 in Table 1). Bars show the mean and standard error for the number of total scrambled lipids extracted from the five 20 μs-long replica simulations.

Next, we estimated the effect of the Sec61, TRAP, and the Sec61/TRAP complex on the scrambling *thermodynamics* by extracting the energetic barriers for lipid flip–flop. For protein-free membranes, we used AWH to extract the potential of mean force (PMF) profile for a POPC flip–flop (see Methods). For the protein-containing system, the scrambling pathway is complicated and thus not easily defined by a single reaction coordinate. Fortunately, the free energy profile can be estimated from lipid headgroup densities across the membrane due to the spontaneous flip–flops (see Methods). The free energy profiles in Figure 4A demonstrate that in the absence of proteins, the barrier is ≈59.2 ± 2.2 kJ/mol, and thereby, spontaneous flip–flops are extremely unlikely. In contrast, the presence of Sec61 in the membrane decreases this barrier to ≈10.8 ± 0.5 kJ/mol. With TRAP alone, the barrier decreases to ≈17.1 ± 0.1 kJ/mol, whereas the simultaneous presence of both Sec61 and TRAP results in the smallest barrier of ≈9.4 ± 0.3 kJ/mol for POPC lipids. These numbers generally follow the scrambling rates in Table 1; the smaller the barrier, the faster the rate. They are also in line with barriers extracted for other scramblases; 7 kJ/mol for MTCH2^32^ and 16 kJ/mol for VDAC1.^43^

These energetics can be put into perspective following the Eyring model. It links the rate *k* with the free energy barrier of a process *G*^‡^ as . Here, *k*_B_ and *h* are the Boltzmann and Planck constants, and κ is a transmission constant often assumed to be equal to unity. In this framework, the observed decrease of the barrier height by ≈50 kJ/mol results in scrambling an ≈250 million times faster in the presence of Sec61/TRAP. For TRAP, this speed-up factor is estimated to be ≈12 million. While in intact phospholipid membranes scrambling takes place in the time scale of a month^9,61^ (half-life), the observed decrease in the energetic barrier would lead to a half-life in the tens of milliseconds for Sec61 and in the hundreds of milliseconds for TRAP. These values are in line with the rates reported for reconstituted VDAC2 dimers,^43^ and also compatible with our experiments that set an upper limit of ≈10 s for the scrambling process (Figures 1E and 1F).

Simulations also allow us to decompose the free energy profiles into their entropic and enthalpic components (see Methods). Thus, we performed the simulations with the Sec61/TRAP complex at various temperatures from 290 to 330 K with 10 K increments (Set 4 in Table 1). The mean free energy profiles for POPC scrambling extracted from the five replica simulations performed at each temperature are shown in Figure 4B. The temperature increase to 330 K leads to the barrier further decreasing to ≈5 kJ/mol. From the decomposed free energy profile shown in Figure 4C, it is evident that there is entropic gain in the lipids partitioning to the membrane and being able to sample more conformations than in its canonical membrane orientation. The presence of Sec61/TRAP leads to a decrease of the entropic term from ≈76 kJ/mol (see Figure S8 in the SI) to ≈38 kJ/mol (dashed green line in Figure 4C) at the position of the free energy barrier. This is expected, as the credit card mechanism limits the conformations sampled by the lipid significantly in the membrane core as compared to a protein-free flip–flop event. The drop in entropic gain with the inclusion of the protein indicates that it must come with a substantial gain in enthalpy. Indeed, the presence of the Sec61/TRAP complex can lower the enthalpy value at the free energy barrier for lipid flip–flop from ≈135 kJ/mol (see Figure S8 in the SI) to ≈48 kJ/mol (dashed red line in Figure 4C) due to favorable interactions between intramembrane polar residues and lipid head groups.

Unfortunately, the only experiment-based decompositions of flip–flop free energies we could find were extracted at a temperature lower than the main transition temperature of the studied lipids.^11^ For DPPC, values of 100.7 ± 0.3 kJ/mol, 245 ± 10 kJ/mol, and 143 ± 8 kJ/mol were extracted for the free energy and its enthalpic and entropic components, respectively. While these are larger than our results likely due to their membrane being in a gel phase, they capture the same trend of a dominant enthalpic penalty partially compensated by entropic gain.^11^

The effect of temperature on scrambling *kinetics* is captured by the Arrhenius formalism (see Methods). The natural logarithm of the rates observed in the simulations (in μs^–1^) as a function of inverse temperature is shown in the Arrhenius plot in Figure 4D. The measured values nicely fall onto a line, indicating an exponential temperature dependence. Thus, protein-mediated lipid scrambling is well described as an activated process with an activation energy of ≈55.3 kJ/mol obtained from the fit in Figure 4D. The relation with the Arrhenius activation energy and the enthalpic contribution of the Eyring equation reads *E*_Arrh_ = Δ*H* + *RT*, and hence the value of *E*_Arrh_ = 55.3 kJ/mol agrees reasonably well with Δ*H* + *RT* = 48 + 2.6 ≈ 51 kJ/mol.

Unfortunately, few activation energy estimates have been derived from fluid-phase membranes, limiting the comparison of this value with experiments. In DPPC and DMPC vesicles, the values of 122 kJ/mol^9^ and 64 kJ/mol^62^ have been reported, whereas a lower value of 50 ± 5 kJ/mol was reported from experiments on supported lipid membranes.^63^ These values highlight that not only the lipid type but also the employed experimental technique could affect the result. Still, the range of experimental values in lipid-only membranes is in the same ballpark as our value for the protein-containing membrane.

### PC Lipids Are Scrambled Most Efficiently

The currently known lipid scramblases facilitate the bidirectional interleaflet movement of lipids regardless of their type—especially headgroup—yet with varying rates. Earlier experimental work has reported headgroup-independent scrambling of lipids (PC, PE, and PS) by TMEM41B,^28,29^ whereas TMEM16K was found to scramble PE and PC lipids approximately 3-fold faster than PS.^26^ To study lipid headgroup preference of Sec61 and TRAP-mediated scrambling, we performed simulations of Sec61, TRAP, and Sec61/TRAP complexes in a larger membrane that contained equimolar amounts of the three major ER lipid moieties: PC, PE, and PS (Set 2 in Table 1). We analyzed the lipid scrambling as earlier, see Table 1 and Figure 4E.

Unlike for the single-component membranes, where the effect of Sec61 and TRAP were additive, in the multicomponent membrane they show a somewhat antagonistic effect; the scrambling rates of the Sec61/TRAP complex are smaller than for the Sec61 and TRAP separately for PC (*p* = 0.005) and PE (*p* = 0.039), but not for PS (*p* = 0.14). Regarding lipid-selectivity of the proteins, our analysis revealed that all tested complexes scramble lipids with a high preference for PC. The Sec61 complex scrambles PC more than PE (*p* = 0.0006) and PS (*p* < 0.00001), whereas PS and PE are scrambled at a similar rate (*p* = 0.12). TRAP was also found to be a more efficient scrambler of PC than PE (*p* = 0.00002) or PS (*p* = 0.0003), and the rate for PE was also higher than that for PS (*p* = 0.0012). PS is the only studied lipid with a charged headgroup, yet its scrambling rates are of the same order of magnitude as those of PC or PE. This suggests that the anionic PS headgroup does not pose a challenge for the scrambling activity by Sec61 or TRAP as the polar and well-hydrated crevices along the scrambling path serve as good solvents for the anionic PS headgroup. This is in line with our experimental data, which demonstrated the rapid scrambling of both NBD PC and NBD PS by the translocon machinery (Figures 1E and 1F). Still, the trends between the lipid head groups are visible in the volumetric density maps shown in Figures S2 and S3 for Sec61 and for the bundle of helices of the three TRAP subunits, respectively. For both scrambling pathways, PC shows the highest occupancy in the membrane core, with PE especially depleted in the case of Sec61.

While it seems at first that the proteins show selectivity toward some lipid types, it is also possible that the observed trends simply follow lipid flip–flop energetics already present in a protein-free membrane. To study this, we again used biased AWH simulations and extracted the barriers for the flip–flops of PC, PE, and PS lipids in a protein-free POPC membrane. The host membrane was always POPC to isolate the effect of the headgroup of the flipping lipid: a POPE membrane would show tighter packing, thus increasing the free energy barrier, whereas the result in a POPS membrane would likely be affected by counterions. With this approach, we found free energy barriers of 59.2 ± 2.2 kJ/mol, 70.6 ± 2.4 kJ/mol, and 63.5 ± 1.4 kJ/mol, for the flip–flop of PC, PE, and PS, respectively (see Figure S4 in the SI). This trend follows the observed rates in Figure 4E, thus suggesting that the proteins are perhaps not selective toward lipid types but instead universally lower the energetic barrier by a similar magnitude, and the differences observed in protein-free membranes are carried over to protein-containing membranes.

One notable feature in Figure 4E is that all studied complexes scramble fewer lipids in the multicomponent membrane compared to the single-component POPC membrane (Table 1 and Figure 4F). This likely results from the extended interaction times of the charged PS headgroup with some polar residues in the scrambling crevices, which can temporarily block the scrambling process. This is especially true for TRAP, but the total scrambling rates of Sec61 and the Sec61/TRAP complex are also lower in the multicomponent membrane (compare orange bars in Figure 4E with those in Figure 4F). Still, the lateral gate crevice in Sec61 is so wide that a single tightly bound PS lipid cannot entirely block the scrambling activity. Similar slower scrambling of PS lipids was also observed experimentally for TMEM16K.^26^

### Potential Limitations of our Study

The possible methodological limitations of our study are also worth a brief discussion. Here, we resorted to coarse-grained simulation models, which provide adequate statistics for scrambling due to their free energy surfaces being smoother than in their atomistic counterparts,^64^ whereas atomistic models support our findings by providing information on the hydration of the scrambling path as well as membrane thinning.^33^ While the Martini 3 model^51^ was developed to mitigate the issue with excessive protein aggregation,^65^ we still had to use backbone position restraints to prevent collapse of the Sec61/TRAP structure. The choice for a criterion used to detect a lipid flip–flop can also affect the absolute numbers of scrambled lipids (see Figure S6 in the SI), yet the qualitative trends among the studied complexes were robust to our choice. Experimentally, while we were able to isolate the ribosome-bound translocon complexes and reconstitute them into LUVs, conclusively assigning the scrambling activity to specific translocon-associated proteins based on experiments alone is not feasible. We note that VDAC1–3 was detectable in the LUVs by mass spectrometry analysis, but quantitative Western blotting reveals that more than 95% of VDACs partition into other fractions. The lack of observed effect of Sec61 inhibitors indicates that another scrambling pathway exists in the translocon complex. Our simulations identified TRAP as a potential candidate, yet despite the success of Martini 3 in predicting scrambling activities of transmembrane proteins,^31,32,43^ further experimental work is required to confirm this finding. Similarly, direct comparison of the scrambling rates between our fluorescence assays and simulations is not feasible (see SI for discussion).

## Conclusions

Here, we report an extensive set of coarse-grained simulations, corroborated by two complementary fluorescence assays, collectively demonstrating that Sec61 translocons scramble lipids in the ER. Our simulations suggest this scrambling to take place along the polar crevices in Sec61 and TRAP via a *credit card mechanism*. Under physiological cellular conditions, the route along the lateral gate of Sec61 is typically either conformationally inaccessible or likely occluded by an inserting nascent polypeptide, and therefore we propose that TRAP could serve as a lipid scramblase regardless of the functional state of Sec61.

In addition to containing polar intramembrane residues, we have earlier demonstrated that Sec61 and TRAP promote local membrane thinning in their vicinity. Our analysis of protein-free membranes suggests that this thinning lowers the free energy barrier of lipid scrambling by up to ≈7 kJ/mol. Considering that the measured barriers are ≈10 kJ/mol and ≈17 kJ/mol for the membranes with Sec61 and TRAP, the effect of thinning plays a significant role in rendering scrambling accessible within thermal fluctuations.

Our free energy decomposition revealed that the presence of the Sec61 and TRAP proteins lowers the free energy barrier by rendering the partitioning of the lipid head groups to the membrane core enthalpically favorable. This is also supported by our atomistic simulations, which highlighted the hydration of the identified scrambling pathways. While the credit card mechanism leads to the loss of conformational entropy compared to a flip–flop in a protein-free system, this is more than compensated by the enthalpic gain.

Finally, we studied the lipid headgroup selectivity of scrambling by Sec61 and TRAP in our simulations. In all cases, PC lipids were scrambled most efficiently. However, our free energy calculations in a protein-free case revealed that the proteins do not show bias toward any headgroup but rather universally lower the energetic barrier for flip–flop. Still, long residence times of charged PS head groups along the scrambling pathway can lower the overall scrambling rates as compared to PC-only membranes.

Our simulations identify and characterize two novel scrambling pathways in the translocon complex in the ER, where scrambling is essential for membrane expansion. Recently, the Sec61 translocon—along with different protein insertases—has been suggested to act as a scramblase.^31^ Similar hypotheses have earlier been made regarding the bacterial SecYEG and yeast Sec61p translocons, yet at least in bacterial inner membranes and in yeast ER membranes their role in scrambling seems redundant, indicating the presence of (also) other scramblases.^40,41^ Moreover, the activity of Sec61 heavily depends on the conformation of its lateral gate and the occupancy of the open conformation with an empty Sec61 channel is expected to be low. Our simulations also revealed that the scrambling by Sec61 is sensitive to the ionic environment, as the inclusion of NaCl led to a significant reduction in the scrambling rate. A detailed analysis revealed that in this case, scrambling takes place almost exclusively along the TRAP pathway. Therefore, TRAP—as a nearly stoichiometric partner of Sec61 in the translocon—could provide a more efficient scrambling pathway available in a physiological setting regardless of the specific functional state of the translocon. Our fluorescence experiments agree with the simulations, as blocking the Sec61 pathway did not eliminate scrambling activity in LUVs, indicating that an alternative pathway—possibly that of TRAP—is active in the reconstituted translocons. Although our findings call for further experimental validation, the inherent challenge in generating purified and reconstituted Sec61,^66^ let alone the Sec61/TRAP complex, precludes this. Instead, our work provides novel insight into the mechanism, kinetics, thermodynamics, and lipid-selectivity of scrambling by the translocon, and suggests TRAP to play a role in lipid scrambling in the ER.

## Experimental Section

### Coarse-Grained Simulations

#### Simulation Systems

We generated two sizes of membranes containing different protein complexes: Sec61, TRAP, Sec61/TRAP, TRAPα, TRAPβ, TRAPγ, or TRAPδ. First, Sec61, TRAP, or the Sec61/TRAP were embedded in smaller single-component membranes made up of POPC (Set 1 in Table 1). These membranes contained 600 POPC lipids, 40 water beads per lipid (total of 24000), and counterions (Cl^–^ or Na^+^) to neutralize the excess protein charge. Additionally, the membrane with Sec61/TRAP was also simulated in the presence of ≈150 mM of NaCl.

Second, Sec61, TRAP, or the Sec61/TRAP were also embedded in larger multicomponent membranes whose leaflets both originally contained 900 lipids with phosphatidylcholine (PC), phosphatidylethanolamine (PE), and phosphatidylserine (PS) lipids present at equal amounts (Set 2 in Table 1). The choice not to mimic the exact composition of the ER membrane^1^ was justified on the proper sampling of the flip–flop events of these different lipid types in the simulation time scale; very few lipids might not diffuse to the protein complex often enough for flip–flops to happen at statistically significant amounts. The membranes were solvated by 35 water beads per lipid for a total of 63000 beads. 600 Na^+^ ions were included to neutralize the charges of the PS head groups, and additional Na^+^ or Cl^–^ ions were included to neutralize the excess charge of the protein complex present. These larger multicomponent membranes were also simulated in the presence of individual TRAP subunits (Set 3 in Table 1). In all simulated membranes, the lipids were modeled to contain palmitate and oleate acyl chains.

The systems were modeled using the latest version 3 of the Martini force field.^51^ The systems were generated using CHARMM-GUI and equilibrated following the CHARMM-GUI protocol.^67^ Then, a 1 μs-long simulation was performed in which the membrane temperature was set to 400 K for fast lipid mixing. The lipid head groups were restrained in the direction normal to the membrane to prevent any flip–flops at this stage. Five frames, separated by 200 ns of simulation time, were extracted and used as initial configurations for the five 20 μs-long production simulations at 310 K. In these simulations, the backbone of the entire protein complex was restrained.

The Sec61/TRAP complex in the smaller single-component POPC membrane was also simulated at multiple temperatures, namely 290, 300, 310, 320, and 330 K (Set 4 in Table 1). Again, five 20 μs-long replicas were performed at each temperature. Here, the goal was to study the activation energy as well as the free energy components of the flipping process, and a smaller membrane with less fluctuations resulted in a more well-defined density profile from which the free energy profile was estimated.

Additionally, we performed simulations of the Sec61/TRAP system with the TRAPα and TRAPγ subunits restrained only at the ribosomal anchoring sites, namely between W255 and K266 of TRAPα as well as between R110 and K115 of TRAPγ, constituting the TRAPα anchor and TRAPγ finger, respectively.^35^ The Sec61 simulation was also repeated without any restraints. The elastic network approach was used in simulations in which the entire protein was not restrained to maintain the tertiary structure. For Sec61, the simulations with the elastic network and no positional restraints, the Sec61 subunits did not remain correctly associated. For simulations of Sec61/TRAP, the TM domains of the latter collapsed onto the Sec61 structure. While lipids were still scrambled by these flawed protein conformations, we have not included the related results in the manuscript.

The free energy barriers for lipid flip–flop in protein-free membranes were calculated using a simulation system containing a total of 201 lipids (100 per leaflet + one that was biased to perform a flip–flop). We performed such simulations for 1) a POPC membrane at 300, 310, and 320 K; 2) a series of PC phospholipids with saturated acyl chains of different lengths, DPTC (2 beads per acyl chain), DLPC (3 beads), DPPC (4 beads), and DBPC (5 beads); 3) a series of lipids with palmitoyl and oleoyl acyl chains and PC, PE, or PS head groups. In the latter case, the membrane consisted of POPC lipids, and only the identity of the flipping lipid varied (POPC, POPE, or POPS).

The Sec61 structure and topology containing all the resistance mutations (I41, D60, M65, R66, S71, G80, S82, T86, Y131, and M136) for the different inhibitors^59^ at once was generated in CHARMM-GUI.^67^

#### Simulation Parameters

The simulations were performed using the SYCL implementation of GROMACS v. 2023 on AMD GPUs.^68,69^ The equations of motion were integrated with the leapfrog integrator with a time step of 25 fs. Buffered Verlet lists were used to keep track of neighboring beads.^70^ Reaction field electrostatics, with a dielectric constant of 15 within the cutoff of 1.1 nm and ∞ beyond it, was used, following the recommended parameters.^52^ The Lennard-Jones potential was shifted to zero at a distance of 1.1 nm. The temperatures of the protein, lipids, and solvent were separately maintained at 310 K with the stochastic velocity rescaling thermostat^71^ with a time constant of 1 ps. The pressure was maintained at 1 bar using the Parrinello–Rahman barostat^72^ with semi-isotropic coupling (two dimensions along the membrane plane coupled together), a time constant of 12 ps, and a compressibility of 3 × 10^–4^ 1/bar.

The free energy barrier for lipid flip–flop in the protein-free membranes was extracted using the accelerated weight histogram (AWH) technique.^73^ Apart from AWH settings, we used identical simulation parameters as for the protein-containing simulations. For AWH, we set the reaction coordinate to be the distance between the headgroup phosphate bead of a single lipid and the rest of the lipid bilayer. The distance was measured along the *z* axis, i.e., along the direction normal to the bilayer. The range of [−4.5, 4.5] of this reaction coordinate was sampled, which resulted in the lipid performing multiple flip–flops during the AWH simulation. When it comes to the AWH options, we used the geometry direction. The convolved potential shape was used, and the two-stage approach was used for rapid convergence. The force constant was set to 10^5^ kJ/(mol × nm^2^). The target coordinate distribution was set to uniform (“constant”). All AWH simulations were 5 μs long.

#### Simulation Analyses

##### Flip–Flop Detection

The flip–flops were characterized by events when a lipid changed its orientation from that characteristic of the upper leaflet to that characteristic of the lower leaflet or *vice versa*. These orientations were defined by a vector connecting the phosphate bead (“PO4”) and the last bead of one of the acyl chains (“C4B”); if the *z* coordinate of the PO4 bead was 2.1 nm smaller (larger) than that of C4B, the lipid was assigned to the upper (lower) leaflet. The numbers of flip–flops visually counted in simulations with individual TRAP subunits (such a visual observation was possible for these systems due to their small number of flip–flops) were reproduced with threshold values of 2.1–2.4 nm. The flip–flop numbers in the single-component membranes with Sec61 or TRAP (Set 1 in Table 1) were also found to plateau around these values, as demonstrated in the top panel of Figure S6 in the SI. Moreover, the difference between Sec61 and TRAP scramblase activity was found to be maximal at the lower end of the range (bottom panel of Figure S6). Thus, we used a threshold value of 2.1 nm in all analyses. Events where a lipid changed its assigned leaflet from one another were recorded as flip–flops.

In the Sec61/TRAP systems, flip–flops were assigned to the protein complex (Sec61 or TRAP) if they initiated within 2 nm of the corresponding scrambling pathway.

##### Activation Energies

The activation energies (*E*_*A*_) for the flip–flop process were extracted with Arrhenius analysis from the flip–flop rates (*k*_flip–flop_) as
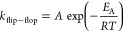
1where *A* is a temperature-independent prefactor, *R* is the universal gas constant, and *T* is temperature.

##### Free Energy Profiles from Unbiased Simulations

The free energy profiles for lipid flip–flop in protein-containing systems were estimated from the density profiles of the phosphate (“PO4”) bead as Δ*G*=–RTln(ρ(*z*) /ρ_0_), where ρ(*z*) is the local density along the *z* axis (normal to the membrane), and ρ_0_ an arbitrary scaling factor chosen so that Δ*G* = 0 in the equilibrium position of PO4 in the membrane. For the density profiles, the system was divided into 300 slices. Since the membrane shape fluctuates, we set a reference to the protein frame. For the trimeric TRAP bundle, we used residues T149 to A173 of TRAPδ, i.e. its transmembrane helix. For Sec61, we used the helical region of TM3, spanning from K97 to M123. For the Sec61/TRAP complex, we used the union of these two groups.

##### Free Energy Decomposition

The entropic and enthalpic components of the flip–flop process were evaluated assuming a constant entropy component between two pairs of temperatures: 300 and 320 K (Δ*T* = 20 *K*) as well as 290 and 330 K (Δ*T* = 40 *K*) using^74^
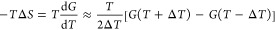
2and
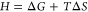
3

For the POPC membrane without Sec61/TRAP, similar analysis was performed using only one pair of temperatures, namely 300 and 320 K.

##### Membrane Thickness

Membrane thickness values were calculated using g_lomepro(75) and based on the local interleaflet distance of the phosphate (“PO4”) beads.

### All-Atom Simulations

We also simulated the Sec61/TRAP complex embedded in a POPC membrane using the all-atom CHARMM36 force field.^76^ The membrane consisted of 400 POPC lipids and was solvated with 100 water molecules per lipid (total of 40000). The excess protein charge was neutralized by Na^+^ ions.

The atomistic system was simulated for 300 ns with an integration time step of 2 fs. Buffered Verlet lists were used to keep track of atomic neighbors.^70^ Long-range electrostatics were implemented using the smooth PME approach.^77,78^ The Lennard-Jones potential was cut off at 1.2 nm, and the forces were switched to zero between 1.0 and 1.2 nm. The temperatures of the membrane components (protein and lipids) and the solvent were separately maintained at 310 K using the Nosé–Hoover thermostat^79,80^ with a time constant of 1 ps. The pressures normal to the membrane plane (*z*) and along it (*x* and *y*) were maintained at 1 bar using the Parrinello–Rahman^72^ barostat with semi-isotropic coupling. The time constant of the barostat was set to 5 ps and the compressibility to 4.5 × 10^–5^ bar^–1^. Bonds involving hydrogens were constrained using P-LINCS,^81,82^ and water geometry was constrained using SETTLE.^83^

### Sample Preparation

#### ER Microsome Isolation and Protein Purification

Sheep pancreatic ER microsomes (SRM) were isolated according to previously described methods.^42,84,85^ The isolated microsomes were treated with micrococcal nuclease in the presence of 1 mM CaCl_2_ to convert polysomes into monosomes.

For the purification of the Sec61/TRAP/ribosome complex, 1 mL of SRM (monosomes) was thawed on ice for 30 min. SRM was solubilized with 1% DDM for 60 min on ice with occasional mixing. The solubilized material was centrifuged at 21,000 × g and further purified by centrifugation at 210,000 × g for 3 h at 277 K through a 10–50% sucrose gradient in 25 mM HEPES (pH 7.4), 125 mM KAc, 15 mM MgCl_2_, 1 mM DTT, and 0.03% DDM. The gradient was then processed using a BioComp Piston Gradient Fractionator to split the gradient into fractions while collecting A_260_. Twelve fractions containing approximately 1 mL were collected, and the A_260_ absorbance of the total fraction was measured using a nanodrop spectrophotometer. The final concentration of the sample was estimated using the molar extension coefficient of eukaryotic ribosomes similar to the earlier study.^42^

#### Large Unilamellar Vesicles

Large Unilamellar Vesicles (LUVs), composed of 99.6% (mol) POPC and 0.4% (mol) of 16:0–12:0 NBD PS; or 0.4% (mol) of 16:0–06:0 NBD PC (Avanti), were prepared by extrusion. Chloroform solutions of POPC and NBD labeled PC or PS were mixed in a round-bottom test tube and dried under a mild flow of nitrogen for 10 min. The lipid film contained 1.514 mg of POPC and 0.006 mg of NBD PC or 0.007 mg of NBD PS. After that, dry lipids were incubated under vacuum overnight. Next day, lipids were hydrated in 500 μL of SECb buffer (50 mM Hepes, 300 mM KAc, 10 mM MgCl_2_, pH 7.5) for 30 min at room temperature and vortexed for 1 min. Multilamellar liposomes, present in the solution after vortexing, were extruded through a 200 nm Nuclepore Track-Etched Membrane, using Avanti Mini Extruder (31 passes through extruder). The final total lipid concentration of LUV sample was 4 mM.

#### Protein Reconstitution

For protein reconstitution in the membrane, we followed a procedure of Brunner and Schenck.^86−88^ Specifically, LUVs were mixed with SEC buffer and 10% (*v*/*w*) Triton X-100 to final concentrations of 3 mM total lipid and 0.19% (*v*/*w*) detergent. This mixture was incubated for 20 min at room temperature to destabilize the LUVs. Then, the protein was added to a final concentration of 79.49 nM, so the final concentration of total lipid was 1.77 mM. After 1 h incubation at room temperature with a gentle mixing, 150 mg/mL of Bio-Beads SM2 (BioRad) was added. This mixture was incubated overnight and gently mixed at 277 K. In parallel, the negative control was prepared in the same way, containing SECb buffer instead of protein solution. The size distribution of LUVs, containing reconstituted translocon, was controlled using DLS (Zetasizer Nano S, Malvern Panalytical). The hydrodynamic diameters and polydispersity indices (PDIs) are shown in Table S2 in the SI. The lipid concentrations here are theoretical, based on the initial amount used in preparation.

TRAP presence in liposomes was confirmed by Western blotting. After the reconstitution process, proteoliposomes were centrifuged at 50,000 × g at 279 K for 1 h, resuspended in fresh SECb buffer and then centrifuged again at the same conditions. The resulting pellet was used for Western blot analysis to assess the presence of TRAP in the reconstituted liposomes. HEK293T lysate, Sheep pancreatic lysate, SRMs, collected fractions, and pelleted LUVs were analyzed by SDS-PAGE and direct protein stain or Western blot. Samples were sorted through 4–20% Tris-Glycine gradient gels. Total protein was visualized by BioRad Flamingo stain method and scanned on a Sapphire Biomolecular Imager with 532 nm excitation and a 572BP28 nm filter. RPS6, TRAPα, TRAPβ. VDAC1/2/3, and TMEM41B were probed for by Western blot.

### Fluorescence Experiments

#### Dithionite Assay

The dithionite assay was performed at 96 well plate (Nunc MicroWell 96-Well Optical-Bottom, Thermo Scientific) using Infinite M Nano+ (Tecan) plate reader. Samples containing the reconstituted proteins and negative controls were measured in the same run. For the experiment, 9 μL of the reconstitution mixture was added to 191 μL of SECb buffer. Light with a wavelength of 460 nm was used for the excitation, whereas the emission was detected at 535 nm. The experiments were performed at 310.15 K. After temperature equilibration, the fluorescence intensity was measured in 20 or 15 s intervals for at least 180 s to acquire the initial plateau. Then, 30 μL of 0.3 M sodium dithionite was added to all samples including the negative controls. The fluorescence decay curves (*F*(*t*)) were normalized so that their maximum intensity was equal to 1.

For the analysis of fluorescence loss kinetics, the *F*(*t*) curves were fitted by a double exponential,
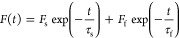
4where *F*_s_ and *F*_f_ are the prefactors of the slow and fast components, respectively. The decay times of these two components are given by τ_s_ and τ_f_. Prior to the fitting, the initial plateaus of the curves in Figure 1E and 1F were removed so that the decay initiated from the point (*t*,*F*(*t*)) = (0,1), hence forcing *F*_s_ + *F*_f_ = 1.

#### BSA Assay

The same setup of the plate reader and the same composition of the initial reaction mixtures, as described for the dithionite assay, was used for the BSA assay. After temperature equilibration and acquirement of an initial plateau, 30 μL of fatty acid-free BSA (50 mg/mL) was added. The fluorescence curves were fitted with eq 4, as for the dithionite assay.

#### Effect of Sec61 Inhibitors

The samples containing inhibitors were measured in parallel with the inhibitor-free samples. Two wells of 96 well plate contained vesicles with the reconstituted translocon and 1 μM inhibitor, and the other two wells contained vesicles with the translocon but in the absence of the inhibitor. The data were fitted using eq 4, as described above.

## Data Availability

All coarse-grained simulation data with protein-containing systems are available online in the Zenodo repository at DOIs: 10.5281/zenodo.10166590, 10.5281/zenodo.10168857, and 10.5281/zenodo.10169434. Experimental data from Fluorescence Assays and Dynamic Light Scattering control measurements are available in the Czech National Repository. DOIs: 10.48700/datst.evhkr-sa820 and 10.48700/datst.hywpj-rhf46. The mass spectrometry proteomics data have been deposited to the ProteomeXchange Consortium via the PRIDE^89^ partner repository with the dataset identifier PXD062310.

## Supporting Information Available

Uncropped gels from Western blotting. Fitting parameters for the fluorescence decay curves. The hydrodynamic diameters and polydispersity indices of the LUVs used in fluorescence assays and in the mass spectrometry. Volumetric maps of lipid densities along the scrambling paths, including for the simulation with salt. Free energy profiles for PC, PE, and PS flip–flop in a POPC membrane. Decomposition of the free energy profiles for a protein-free POPC membrane. Characterization of the effect of the detection threshold on the number of detected flip–flops. List of selected proteins identified by MS. (PDF)
