# Supplementary material for: Lipid Scrambling Pathways in the Sec61 Translocon Complex
Source: J Am Chem Soc. 2025 May 6;147(19):15970–84. doi: 10.1021/jacs.4c11142 (PMC12082634; doi:10.1021/jacs.4c11142)
Supplement: Supplementary file 1 — ja4c11142_si_001.pdf [file ja4c11142_si_001.pdf]

Supporting Information: Lipid Scrambling Pathways in the Sec61 Translocon Complex

Matti Javanainen,[*,†,‡,@] Jan Šimek,[¶,§,@] Dale Tranter,[‡,@] Sarah O'Keefe,[‡] Sudeep Karki,[‡,‖] Denys Biriukov,[⊥,#] Radek Šachl,[*,¶] and Ville O. Paavilainen[*,‡]

†*Unit of Physics, University of Tampere, FI-33720 Tampere, Finland*

‡*Institute of Biotechnology, HiLIFE, University of Helsinki, FI-00790 Helsinki, Finland*

¶*J. Heyrovský Institute of Physical Chemistry, CZ-18223 Prague 8, Czech Republic*

§*Department of Physical and Macromolecular Chemistry, Charles University, Hlavova 8, CZ-12800 Prague 2, Czech Republic*

‖*Onego Bio, Hämeentie 157, FI-00560 Helsinki, Finland*

⊥*Central European Institute of Technology, Masaryk University, Kamenice 5, CZ-62500 Brno, Czech Republic*

#*National Centre for Biomolecular Research, Faculty of Science, Masaryk University, Kamenice 5, CZ-62500 Brno, Czech Republic*

@*These authors contributed equally to this work*

E-mail: matti.javanainen@tuni.fi; radek.sachl@jh-inst.cas.cz; ville.paavilainen@helsinki.fi

Fluorescence Experiments

Comparison of the Scrambling Rates From Experiments and Simulations

We fitted the fluorescence data in Figs. 1E and 1F in the main text with a double exponential (Eq. (4) in Methods). The fitting parameters are listed in Table S1. Overall, the two time constants were in the ranges of 50–110 s and 1000–9000 s, likely corresponding to the BSA-mediated fluorescence quenching or dithionite-mediated chemical reduction of NBD and the eventual dimming of the fluorescent labels over time. For the dithionite assay with NBD PC, the exponential term struggles to capture the rapid initial decay of the fluorescence signal (see Figs. 1E and 1F in the main text). Overall, the smaller of the two time constants, τ_f, is of more interest in the context of scrambling. Its values were consistently larger for the protein-containing samples. At first, one could associate this with the slower loss of fluorescence of the inner leaflet lipids that have to be scrambled to the outer leaflet and only then reduced or quenched. However, it is more likely that τ_f describes the two processes—dithionite diffusion to the LUV surface and its reaction thereon—instead of the significantly faster scrambling process. Indeed, LUVs with a radius of ≈200 nm contain a total of ≈2 million POPC lipids in its two leaflets, and their scrambling at a rate of ≥2/µs would only take ≤1 s by a single Sec61/TRAP complex, assuming that each lipid is scrambled only once. The process is also not diffusion-limited, as with a typical diffusion coefficient of 10^{-8} cm^2/s, a lipid would diffuse to the scrambling site from the other side of the LUV in <1 s. This also indicates that it is challenging to pinpoint the number of Sec61/TRAP complexes in our sample based on the upper limit of ≈10 s for scrambling. The same conclusions are reached following the approach of Mathiassen et al..[S1] Using the average width of the Sec61/TRAP complex, ≈8 nm as the extent of the scrambling site, we obtained $\tau_c = 0.023$ s for LUVs containing only a single scramblase each. Narrowing down the scrambling site radius to 0.5 nm approximately doubles the estimate of τ_c, yet it is still orders of magnitude smaller than the experimentally

recorded time constants for scrambling, indicating that the process is not diffusion-limited. The residual fluorescence of ≈15–20% in our dithionite experiments (Fig. 1E in the main text) could correspond to LUVs not containing a single Sec61/TRAP complex. The slower loss of fluorescence kinetics in the protein-containing sample likely results from the obstruction of the free passage of the quencher to the LUV surface by the reconstituted proteins, especially the ribosome. This especially affects BSA, whose slower quenching kinetics also results from its more complex quenching process and its slower diffusion in the solvent. Evaluating lipid selectivity from experiments is also challenging. NBD PC is quenched or reduced faster than NBD PS regardless of the presence of the translocon (Table S1), which could result from the repulsive interaction of anionic BSA and dithionite with the anionic PC lipids as well as the the longer NBD-attached acyl chain in NBD PS as compared to NBD PC (Fig. 1B).

Table S1: **Fitting parameters from fluorescence experiments.** The double exponential fitting parameters of Eq. (4) to the data in Figs. 1E and 1F.

**Dithionite assay**								
	NBD PC				NBD PS			
Sample	F_f	F_s	τ_f (s)	τ_s (s)	F_f	F_s	τ_f (s)	τ_s (s)
No proteins	0.59	0.36	9.9	2783	0.30	0.62	78	8825
Proteins	0.73	0.24	5.9	1798	0.59	0.31	84	3614
Proteins + AprA	0.71	0.24	5.7	1803	0.57	0.30	86	3738
Proteins + ipomF	0.72	0.24	6.2	1945	0.59	0.30	80	3377
**BSA assay**								
	NBD PC				NBD PS			
Sample	F_s	F_f	τ_s (s)	τ_f (s)	F_s	F_f	τ_s (s)	τ_f (s)
No proteins	0.13	0.82	51	2166	0.19	0.76	49	4332
Proteins	0.33	0.57	62	1238	0.28	0.67	110	1458
Proteins + AprA	0.31	0.55	65	1183	0.32	0.62	95	1327
Proteins + ipomF	0.32	0.55	63	1243	0.32	0.61	109	1524

Supplementary Data

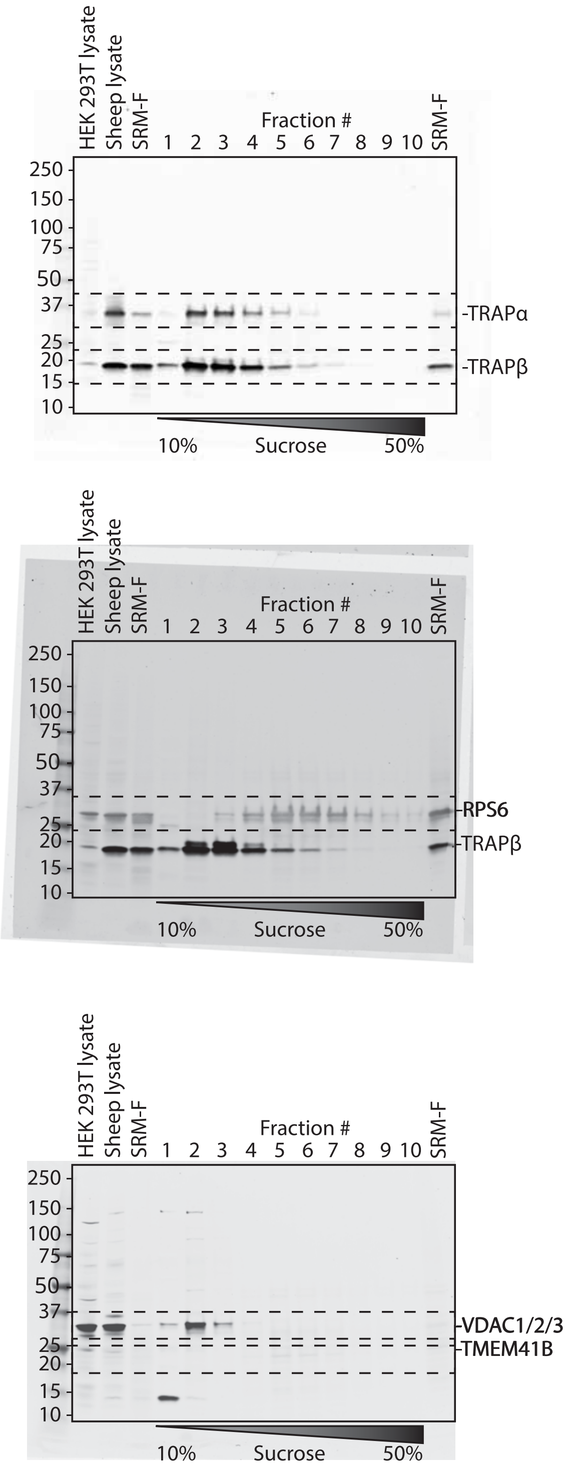

Figure S1: **Uncropped scans of Western blots used in Fig. 1A.** Dashed lines indicate boundaries of the areas cropped to be used in Fig. 1A in the main text.

Table S2: **Particle size and homogeneity control of LUVs before and after the reconstitution procedure.** The particle size (hydrodynamic diameter) and homogeneity (polydispersity index, PDI) were assessed after the reconstitution process for each sample used in the experiments shown in Figure 1. Since DLS measurements indicated the presence of aggregates in the reconstituted samples, we also report DLS data for the LUVs samples prior to reconstitution.

Sample	Hydrodynamic diameter (nm)	PDI
**Dithionite and BSA assays**		
NBD PC after extrusion	173.8 ± 1.050	0.109 ± 0.036
NBD PS after extrusion	175.6 ± 2.651	0.094 ± 0.014
NBD PC + Proteins	349.6 ± 5.027	0.580 ± 0.052
NBD PS + Proteins	393.1 ± 11.89	0.533 ± 0.050
**Western blot**		
NBD PC after extrusion	174.3 ± 5.085	0.075 ± 0.028
NBD PC + Proteins	330.4 ± 32.35	0.767 ± 0.081

Mass Spectrometry

Samples for MS analysis were prepared following the protocol described in the Protein Reconstitution section. After the reconstitution process, the sample was centrifuged at 50,000×g at 279 K. The pellet and the supernatant were divided. The sample for the MS analysis was prepared in a triplicate.

The pellet samples, resuspended in 50 µL of 50 mM ammonium bicarbonate, and the supernatant samples were reduced and alkylated by TCEP and chloroacetamide, and mixed with 10 µL of SpeedBead Magnetic Carboxylate (Cytiva). Ethanol was added to the samples in a 1:1 ratio (v/v). After the 10 min incubation at room temperature, the magnetic beads were washed twice with 200 µL of 80% ethanol and dried at room temperature. Dry beads were resuspended in 50 µL of 50 mM ammonium bicarbonate and digested by trypsin overnight at 310 K. Samples were analyzed using a liquid chromatography system Vanquish (Thermo Scientific) connected to the timsTOF SCP mass spectrometer equipped with Captive spray (Bruker Daltonics). The mass spectrometer was operated in a positive data-dependent mode.

Three microliters of peptide mixture were injected by autosampler on the C18 trap column (Pepmap Neo C18 µm, 0.3×5 mm, Thermo Scientific). After trapping, peptides were eluted from the trap column and separated on a C18 column (Pepsep C18 150×0.15 mm, 1.5 µL, Bruker Daltonics) by a linear 35 min water-acetonitrile gradient from 5% (*v/v*) to 35% (*v/v*) acetonitrile at a flow rate of 1.5 µL/min. The trap and analytical columns were both heated to 323 K. Parameters from the standard proteomics PASEF method were set to timsTOF SCP. The target intensity per individual PASEF precursor was set to 20000, and the intensity threshold was set to 1500. The scan range was set between 0.6 and 1.6 Vs/cm^2 with a ramp time of 100 ms. Number of PASEF MS/MS scans was 10. Precursor ions in the m/z range between 100 and 1700 with charge states ≥2+ and ≤6+ were selected for fragmentation. The active exclusion was enabled for 0.4 min.

The raw data were processed by PeaksStudio 11 software (Bioinformatics Solutions, Canada). The search parameters were set as follows: enzyme – trypsin (specific), carbamidomethylation as a fixed modification, oxidation of methionine and acetylation of protein N-terminus as variable modifications. The data were searched against the *Ovis aries* protein database. The relative quantification was performed using the LFQ method (PeaksStudio 11) with the following parameters: Mass Error Tolerance: 20.0 ppm, Retention time shift tolerance: Auto-detected, Feature intensity ≥ 300.0, Ion Mobility Tolerance (1/k0): 0.05, Retention time range: [0.0 - Max], Significance method: ANOVA, Use Top 3 peptide, Normalization method: TIC.

Additional Simulation Data

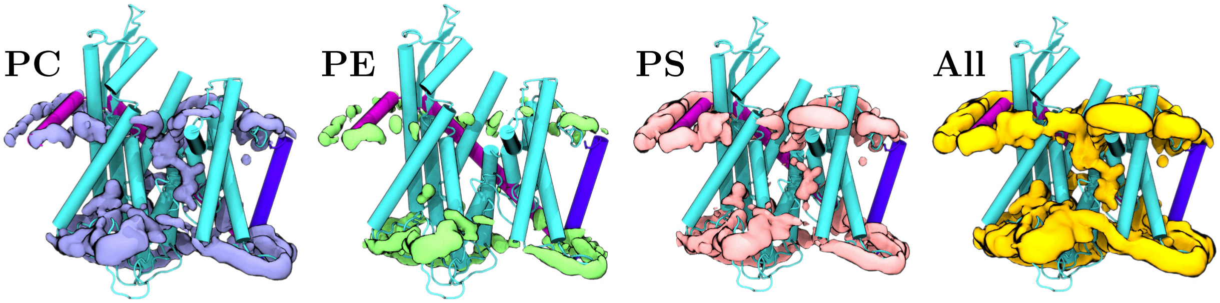

Figure S2: **Volumetric maps of the densities of lipid moieties around Sec61.** The maps are calculated from the simulation with the Sec61/TRAP complex present in the multicomponent lipid membrane. The densities are shown separately for all components, and the map for the total density ("All") is also repeated from the main text for easy comparison. The all-atom structure for the protein is used for visualization purposes. Coloring as in Fig. 2: Sec61α is shown in cyan, Sec61β in blue, and Sec61γ in purple. Lipids are colored as in Fig. 4E. The threshold for the maps was set at 0.01 Å^{-3} in VMD, and all replicas were used in the analysis (5×20 µs).

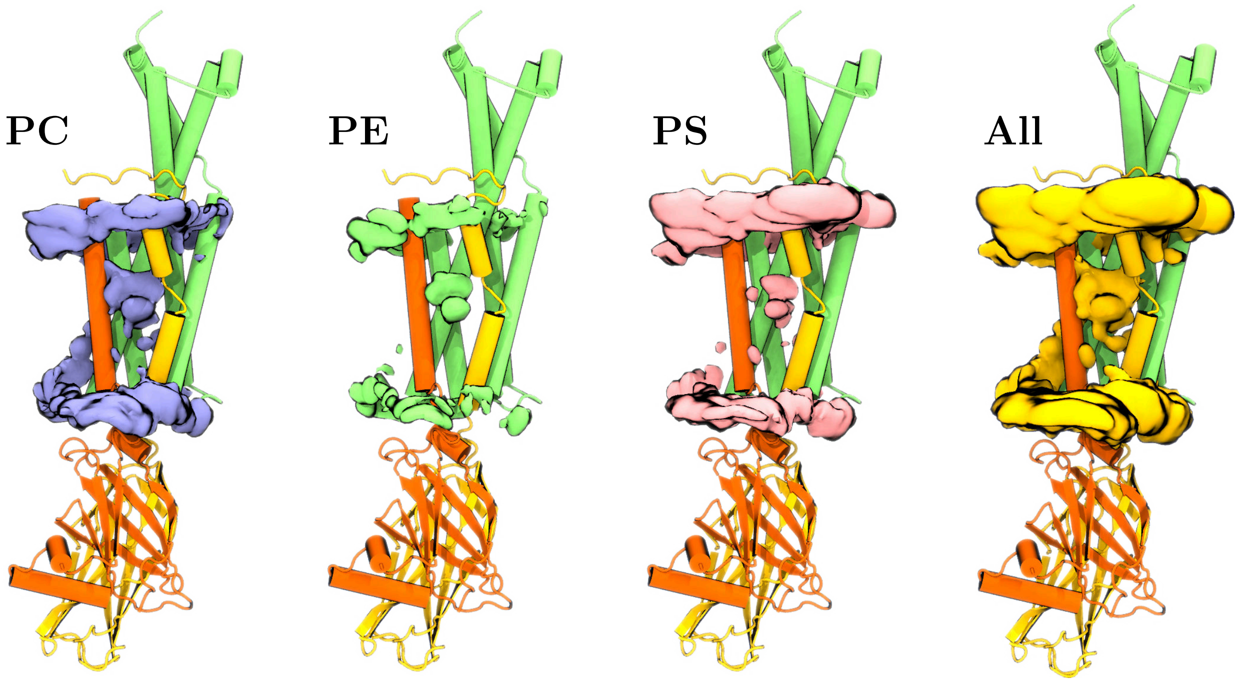

Figure S3: **Volumetric maps of the densities of lipid moieties around the bundle of TRAPβ, TRAPγ, and TRAPδ.** The maps are calculated from the simulation with the Sec61/TRAP complex present in the three-component lipid membrane. The densities are shown separately for all components, and the map for the total density ("All") is also repeated from the main text for easy comparison. The all-atom structure for the protein is used for visualization purposes. Coloring as in Fig. 2: TRAPβ is shown in yellow, TRAPγ in green, and TRAPδ in orange. Lipids are colored as in Fig. 4E. The threshold for the maps was set at 0.01 Å^{-3} in VMD, and all replicas were used in the analysis (5 × 20 µs).

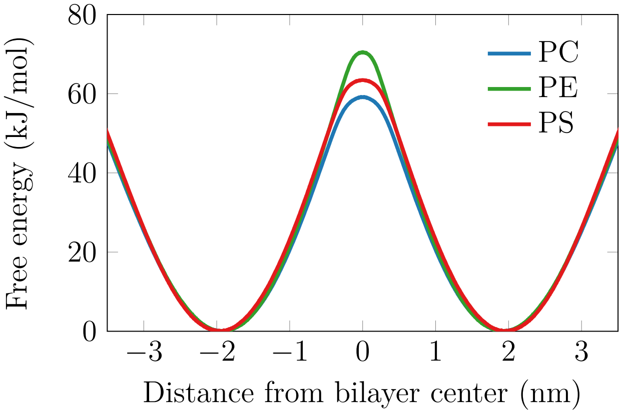

Figure S4: **Free energy profiles for lipid flip–flop in a POPC membrane.** The profiles are extracted for palmitoyloleoylphosphatidylcholine lipids with three different head groups: PE, PC, and PS. The host membrane is POPC. The profiles obtained from the AWH method are symmetrized, and the AWH error estimates are 0.26–0.37 kJ/mol.

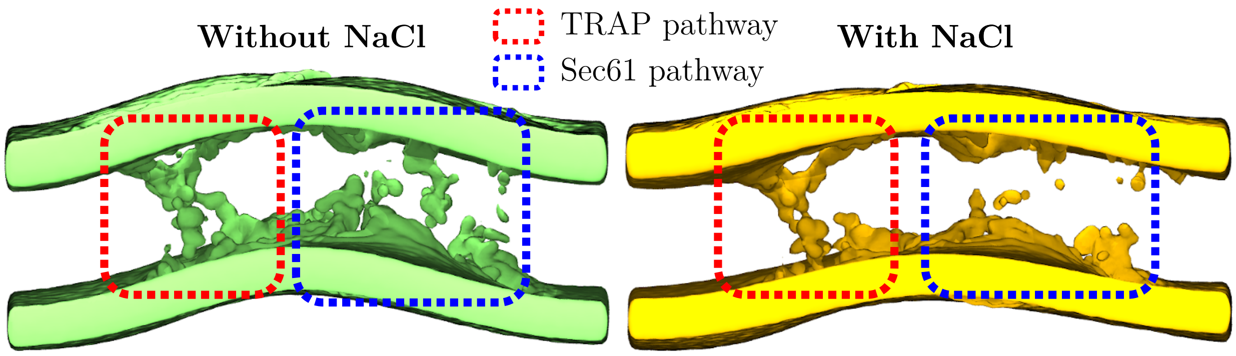

Figure S5: **Effect of NaCl salt on lipid scrambling.** Data are shown for the single-component POPC membrane. The surface presentation shows the volumetric density of the phosphate (PO4) bead of POPC. The threshold for the maps was set at 0.02 Å^{-3} in VMD. The two panels show the data for the system without NaCl (counter-ions to neutralize the charge of protein residues are still included) and with ≈150 mM NaCl. The two pathways are highlighted with colored rectangles. In the presence of salt, the Sec61 pathway is not accessible to the PC head group, whereas the TRAP pathway is unaffected.

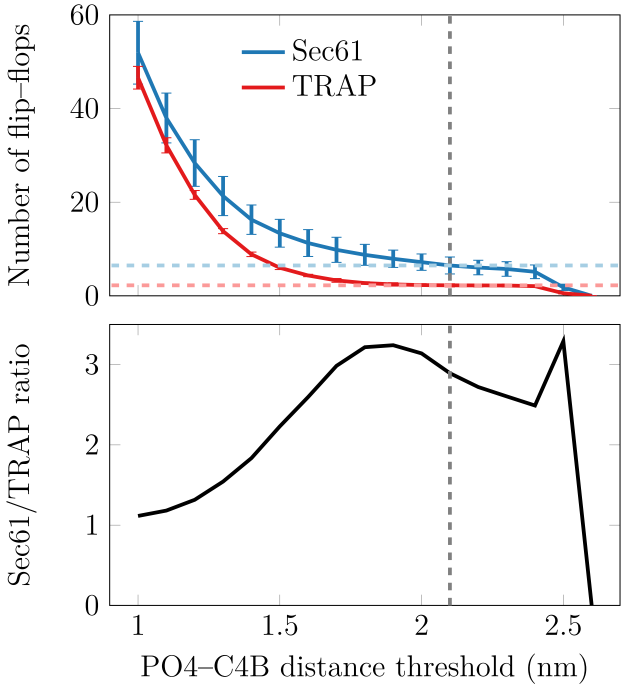

Figure S6: **Effect of detection threshold on the number of observed flip–flops.** Top: The numbers of flip–flops in the single-component membranes (Set 1 in Table 1) for Sec61 and TRAP as a function of PO4–C4B distance threshold used in flip–flop detection. The dashed gray lines show the value of 2.1 nm used in all calculations. The light blue and light red lines highlight that the number of flip–flops is relatively insensitive around the chosen value. Bottom: The ratio of flip–flops in the systems with Sec61 and TRAP. The gray dashed line again shows the chosen value of 2.1 nm.

Table S3: **Relevant hits in the MS analysis of reconstituted LUVs.** For full set of detected proteins, see an SI document (.csv). All Sec61 and TRAP subunits are detected as expected based on the Western blotting (see main text). Additionally, VDAC2 is detected, although Western blotting suggests that it not present in the LUVs at significant concentrations. Unfortunately, MS does not allow for quantitative estimates of protein concentrations due to the different ionization effectivities of their peptides. The data for enrichment between the pellet and supernatant is omitted from this table, as we were unable to properly separate vesicles into the pellet fraction. To demonstrate this, we prepared a sample containing an NBD-labeled lipid following the same protocol as in our experiments. We then measured fluorescence in the initial sample (before centrifugation), the supernatant, the washing buffer, and the final pellet. The results indicate that under these conditions, only approximately 20—30% of the liposomes are pelleted. (See Fig. S7). PG: protein group, Sign.: significance, Cov.: coverage (%), #P: number of peptides, #U: number of unique peptides, PTM: post-translational modifications, Acet.: acetylation, Ox.: oxidation, Carb.: carbamidomethylation

**Accession**	**Sign.**	**Cov.**	**#P**	**#U**	**PTM**	**Description**
W5PP18_SHEEP	10.24	42.65	4	4	Acet. Ox. (M)	Protein transport protein Sec61 subunit gamma OS=Ovis aries OX=9940 PE=3 SV=1
W5P5C3_SHEEP	4.98	25.44	14	8	Ox. (M)	SEC61 translocon subunit alpha 1 OS=Ovis aries OX=9940 GN=SEC61A1 PE=3 SV=1
W5QGC4_SHEEP	11.36	15.62	1	1		Protein transport protein Sec61 subunit beta OS=Ovis aries OX=9940 PE=3 SV=1
W5P572_SHEEP	3.07	53.76	5	5		Translocon-associated protein subunit beta OS=Ovis aries OX=9940 GN=SSR2 PE=3 SV=1
W5NYA9_SHEEP	10.92	44.86	7	7		Translocon-associated protein subunit gamma OS=Ovis aries OX=9940 GN=SSR3 PE=3 SV=1
W5Q9F1_SHEEP	5.42	41.79	8	8	Ox. (M)	Translocon-associated protein subunit alpha OS=Ovis aries OX=9940 PE=3 SV=1
W5P940_SHEEP	23.27	41.62	8	8	Ox. (M)	Translocon-associated protein subunit delta OS=Ovis aries OX=9940 GN=SSR4 PE=3 SV=1
W5PG36_SHEEP	5.81	57.14	12	12	Carb.	Voltage-dependent anion-selective channel protein 2 OS=Ovis aries OX=9940 GN=VDAC2 PE=3 SV=1

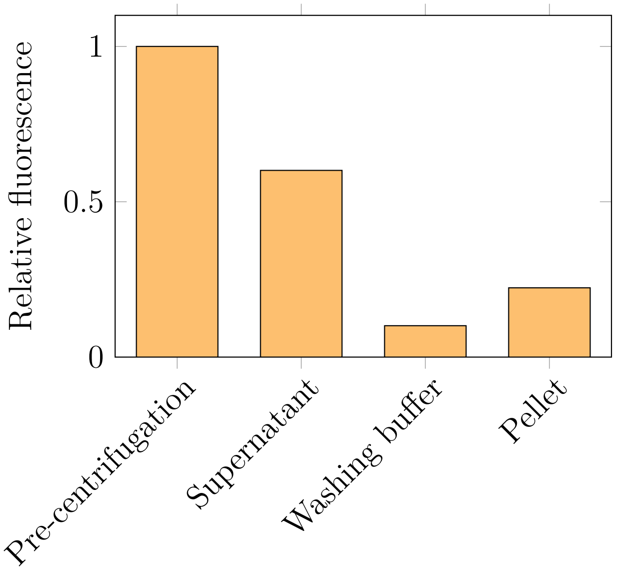

Figure S7: Comparison of fluorescence intensities of sample before centrifugation, supernatant, washing buffer, and pellet.

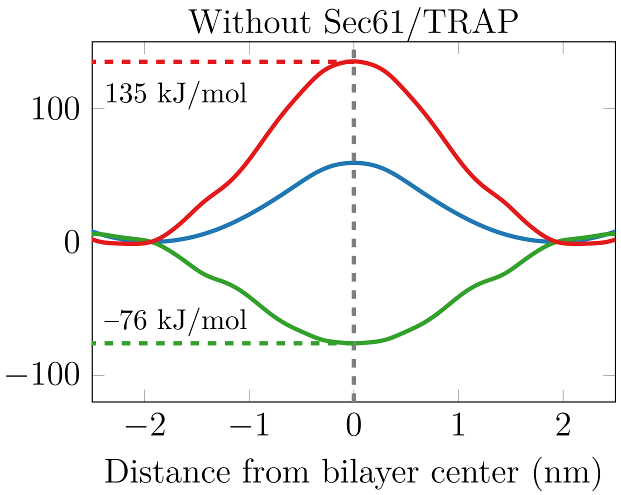

Figure S8: Free energy decomposition in the protein-free POPC membrane. The entropic and enthalpic components at the free energy barrier are highlighted. Coloring as in Fig. 4C in the main text.

References

(S1) Mathiassen, P. P.; Menon, A. K.; Pomorski, T. G. Endoplasmic Reticulum Phospholipid Scramblase Activity Revealed After Protein Reconstitution Into Giant Unilamellar Vesicles Containing a Photostable Lipid Reporter. *Sci. Rep.* **2021**, *11*, 14364.
